# Bioprospecting for Anti-Kinetoplastid Drug Discovery from *Aloysia citrodora* Essential Oil

**DOI:** 10.3390/ijms26125697

**Published:** 2025-06-13

**Authors:** Amani Omrani, Meriam Ben Youssef, Ines Sifaoui, Eduardo Hernández-Álvarez, María J. Trujillo-Rodríguez, Montse Saura-Cayuela, Verónica Pino, Hichem Sebai, Isabel L. Bazzocchi, Jacob Lorenzo-Morales, José E. Piñero, Ignacio A. Jiménez

**Affiliations:** 1 Instituto Universitario de Enfermedades Tropicales y Salud Pública de Canarias, Universidad de La Laguna, 38296 San Cristóbal de La Laguna, Santa Cruz de Tenerife, Spain; alu0101534855@ull.edu.es (A.O.); alu0101534848@ull.edu.es (M.B.Y.); isifaoui@ull.edu.es (I.S.); mtrujill@ull.edu.es (M.J.T.-R.); msauraca@ull.edu.es (M.S.-C.); veropino@ull.edu.es (V.P.); jmlorenz@ull.edu.es (J.L.-M.); jpinero@ull.edu.es (J.E.P.); 2 Laboratory of Functional Physiology and Valorization of Bio-Ressources, Higher Institute of Biotechnology of Beja, University of Jendouba, Beja 382-9000, Tunisia; sebaihichem@yahoo.fr; 3 Instituto Universitario de Bio-Orgánica Antonio González and Departamento de Química Orgánica, Universidad de La Laguna, Avenida Astrofísico Francisco Sánchez 2, 38206 San Cristóbal de La Laguna, Santa Cruz de Tenerife, Spain; alu0100947311@ull.edu.es (E.H.-Á.); ilopez@ull.edu.es (I.L.B.); 4 Departamento de Obstetricia y Ginecología, Pediatría, Medicina Preventiva y Salud Pública, Toxicología, Medicina Legal y Forense y Parasitología, Universidad de La Laguna, C/Sta. María Soledad s/n, 38200 San Cristóbal de La Laguna, Santa Cruz de Tenerife, Spain; 5 Consorcio Centro de investigación Biomédica en Red, Área de Enfermedades Infecciosas (CIBERINFEC) Instituto de Salud Carlos III, Av. Monforte de Lemos 3–5, Pabellón 11, 28029 Madrid, Spain; 6 Departamento de Química, Unidad Departamental de Química Analítica, Universidad de La Laguna (ULL), 38206 San Cristóbal de La Laguna, Santa Cruz de Tenerife, Spain

**Keywords:** *Aloysia citrodora*, essential oil, anti-kinetoplastid activity, bioassay-guided fractionation, chemical modification, mechanism of action

## Abstract

Natural products have long been recognized as invaluable resources in drug discovery. Essential oils have attracted widespread attention due to their broad spectrum of biological activities. Herein, we report the anti-kinetoplastid activity of *Aloysia citrodora* leaf essential oil through a bioassay-guided fractionation method against the etiological agents of Chagas disease and leishmaniasis. This approach has led to the isolation and structural identification of compound **1** (citral) as the main active constituent, with IC_50_ values of 8.47 μM against *Leishmania amazonensis* and 12.90 μM against *Trypanosoma cruzi*. In addition, eight compounds (**2**–**9**) were synthesized and evaluated. Among these, citral 2,4-dinitrophenylhydrazone (**9**) exhibited the highest anti-kinetoplastid activity, with an IC_50_ value of 10.62 μM against *L. amazonensis*, displaying a similar biological profile to citral and the reference drug. Structure–activity relationship studies revealed that the type of Schiff base and acylating agent played a crucial role in the activity. Mechanism of action studies demonstrated that compound **9** directly targets the apoptotic pathway, inducing programmed cell death through selective pathway inhibition. This work underscores the potential of *A. citrodora* essential oil and its compounds as prospective therapeutic leads against neglected tropical diseases.

## 1. Introduction

Parasitic diseases pose a significant global health challenge, particularly in developing countries [1]. According to recent estimates, over 60% of the population in endemic regions is affected by various parasitic infections [2]. These diseases not only result in substantial morbidity and mortality but also impose severe economic burdens and hinder development in the affected areas [1,2].

Leishmaniasis and Chagas disease comprise a diverse group of infectious diseases caused by the protozoan parasites *Leishmania* [3,4] and *Trypanosoma cruzi* [5,6], which disproportionately affect tropical and subtropical regions. The burden of these neglected tropical diseases is highest in lower-middle-income and low-income countries due to factors such as malnutrition, poverty, climate change, and limited access to healthcare and social protection systems [7]. The epidemiology of these diseases is complex and often linked to environmental conditions. Many of them are vector-borne, have animal reservoirs, and involve intricate life cycles, making public health control efforts particularly arduous. Currently, in the absence of a vaccine, no highly effective or specific therapeutic treatments are available for these infections. Current chemotherapy presents several drawbacks, including high costs, toxicity, and complex treatment regimens, which can lead to severe side effects and the emergence of drug-resistant parasites [8]. Therefore, the development of new drugs is crucial to preventing and combating these major parasitic infections.

Natural products have played a pivotal role in the discovery and development of contemporary therapeutic agents, particularly in the fields of oncology and infectious diseases [9,10]. The rich diversity of chemical structures found in nature has provided an invaluable source of bioactive compounds, many of which have become cornerstone treatments in modern medicine. Recent studies have highlighted the significance of essential oils (EOs) in the drug discovery pipeline. They are complex mixtures, typically volatile and odorous, and composed mainly of monoterpenoids and sesquiterpenoids [11]. These plant-derived compounds have shown a broad spectrum of biological activities, including antimicrobial, antifeedant, and antiviral properties [11,12]. Interestingly, a growing body of evidence indicates that numerous essential oils and their bioactive constituents exhibit significant anti-kinetoplastid and antiparasitic activity, highlighting their potential as promising candidates for the development of novel therapeutics against parasitic diseases. Notably, essential oils derived from *Artemisia annua* [13], *Origanum vulgare* [14], *Eugenia caryophyllata* (*Syzygium aromaticum*) [15], and *Thymus vulgaris* [16] have demonstrated potent in vitro activity against various *Leishmania* and *Trypanosoma* species. These antiparasitic effects are primarily attributed to their major components, such as artemisinin, carvacrol, eugenol, and thymol. They act by inducing oxidative stress, disrupting cellular membranes, and impairing mitochondrial function in the parasites. These findings underscore the need for comprehensive studies to elucidate the chemical profiles, pharmacodynamics, and molecular targets of essential oil constituents, which will be crucial for clarifying their mechanisms of action and advancing their development as effective anti-kinetoplastid agents.

*Aloysia citrodora* (*Verbenaceae*), commonly known as “lemon verbena”, has been widely used in herbal teas due to its digestive, antispasmodic, anti-inflammatory, antioxidant properties and for treating respiratory and gastrointestinal disorders [17,18]. Furthermore, “lemon verbena” essential oil has a history of traditional medicinal use due to its antioxidant and antimicrobial properties, offering potential health benefits [19,20]. The main components of “lemon verbena” EOs are geranial, neral, limonene, and spathulenol, all of which exhibit a broad range of biological activities [21].

To continue our efforts towards the discovery of novel plant-derived antiparasitic agents, *A. citrodora* was selected for investigation based on its traditional use and reported bioactivity. In this study, we aim to evaluate the antiparasitic potential of its essential oil (EO) against the globally relevant kinetoplastid pathogens *Leishmania amazonensis* and *Trypanosoma cruzi* using a bioactivity-guided fractionation strategy. Our primary objectives are to isolate and identify the major bioactive constituents responsible for the observed activity, assess their cytotoxicity, and explore structure–activity relationships through the synthesis and evaluation of citral derivatives. The most promising compound was subsequently subjected to mechanistic studies to elucidate its mode of action, focusing on its effects on membrane integrity, oxidative stress induction, chromatin alterations, and mitochondrial function. Overall, this work contributes to the ongoing search for effective and low-toxicity antiparasitic agents from natural sources by providing valuable insights into the potential of *A. citrodora* and its constituents as promising leads for the development of new antiparasitic therapies.

## 2. Results and Discussion

### 2.1. Bioassay-Guided Fractionation

**Figure 1:**
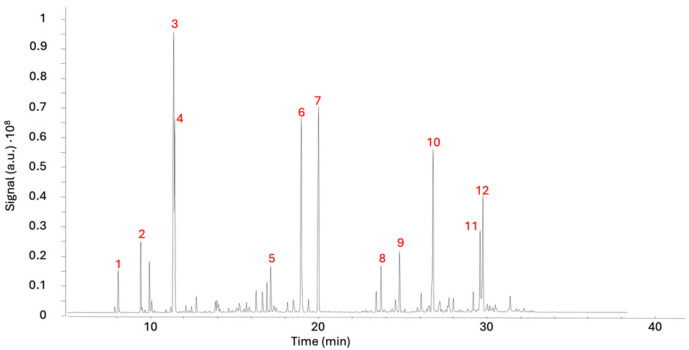
GC-MS chromatogram of *A. citrodora* essential oil. Note: Compounds identified (**1**–**12**) are listed in Table 1.

The most common approach in drug discovery for identifying active compounds involves extensive screening of crude natural extracts using bioassay-guided protocols [22]. In this context, the EO from *Aloysia citrodora* leaves was evaluated against *Leishmania amazonensis* promastigotes and *Trypanosoma cruzi* epimastigotes. For comparative purposes, miltefosine and benznidazole, the reference drugs for leishmaniasis and Chagas disease, respectively, were also evaluated. Miltefosine exhibited an IC_50_ of 2.64 μg/mL against *L. amazonensis*, while benznidazole demonstrated an IC_50_ of 1.81 μg/mL against *T. cruzi*. The results of the anti-kinetoplastid assays revealed that the EO exhibited activity against both tested strains and showed a strong effect against *L. amazonensis* with an IC_50_ value of 10.67 μg/mL and moderate activity against *T. cruzi* with an IC_50_ of 22.6 μg/mL. Furthermore, the EO was screened for its safety profile on the murine macrophage cell line J774.A1 and showed a CC_50_ of 51.6 ± 0.95 μg/mL. In light of these findings, the volatile and semi-volatile profiles of the EO were obtained using gas chromatography–mass spectrometry (GC/MS). This preliminary step enabled the identification and relative quantification of the major volatile and semi-volatile constituents. A summary of the analytical results is presented in Figure 1 and Table 1. At this stage, the study focused on the most abundant compounds observed in the chromatogram, which represent 76% of the total separated constituents (Figure 1). Identification was carried out using the NIST MS library and via the Kovats indexes. The major constituents were D-limonene (16.0%), citral (11.8%), neral (11.1%), α-curcumene (8.71%), eucalyptol (6.91%), and caryophyllene oxide (6.34%). Minor constituents were not identified due to poor spectral quality. The GC-MS data provided critical insights into the oil’s composition, facilitating a more rational and informed selection of chromatographic conditions.

These observations identify the genus *Aloysia* as a promising source for the development of therapeutic agents against kinetoplastid diseases, including leishmaniasis and trypanosomiasis. It highlights the importance of recent research on the antiparasitic activity of the *Aloysia* species, particularly noting that *Aloysia gratissima* has demonstrated significant efficacy against *Leishmania amazonensis*. The essential oil of *A. gratissima*, along with its major sesquiterpene, guaiol, has also exhibited potent activity against both promastigotes and intracellular amastigotes while showing minimal toxicity to macrophages [23]. Additionally, this research is the first investigation into the activity of *Aloysia* species against *Trypanosoma* parasites, further emphasizing their potential as sources of antiparasitic agents.

**Figure 2:**
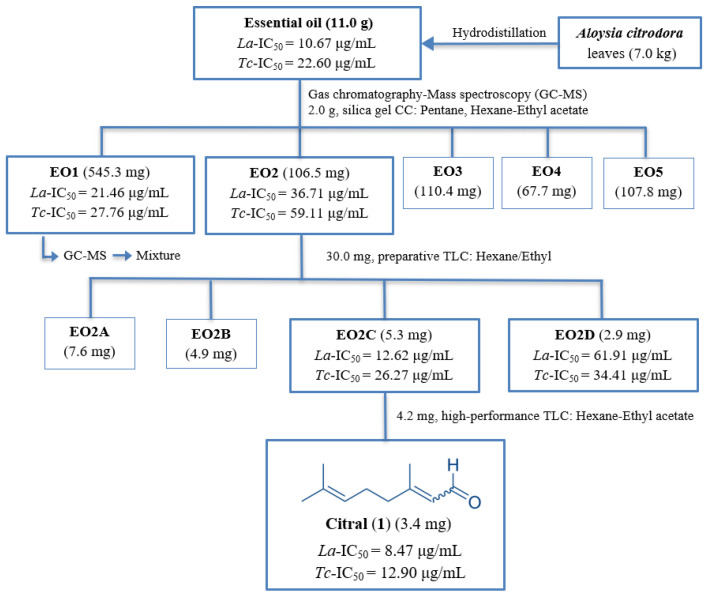
Flowchart of antiprotozoal bio-guided fractionation of *Aloysia citrodora* leaf EO against *L. amazonensis* (*La*) promastigotes and *T. cruzi* (*Tc*) epimastigotes. IC_50_: The concentration able to inhibit 50% of the growth of the tested parasite (µg/mL).

These findings led us to conduct a phytochemical investigation using bioassay-guided fractionation. Consequently, the EO was subjected to silica gel CC to afford five fractions (EO1–EO5). Among these, fractions EO1 and EO2 exhibited promising in vitro anti-kinetoplastid activity, the fraction EO1 showed an IC_50_ of 17.38 μg/mL against *L. amazonensis* and 24.35 μg/mL against *T. cruzi* and the fraction EO2 showed an IC_50_ of 33.83 μg/mL against *L. amazonensis* and 55.59 μg/mL against *T. cruzi*. This encouraged us to conduct further studies to elucidate their chemical composition (Figure 2 and Table 2).

**Figure 3:**
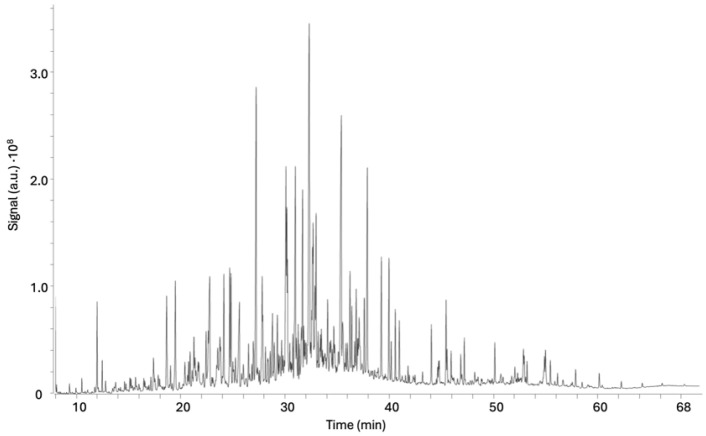
GC-MS chromatogram of fraction EO1.

Thus, the active fraction EO2 (30 mg), characterized by its relatively simpler composition, was purified by two step of silica gel preparative thin-layer chromatography (TLC) using the hexane–ethyl acetate mixture (9:1) to give compound **1**, which was identified as citral (3,7-dimethyl-2,6-octadienal), a naturally occurring mixture composed of two geometric isomers of acyclic monoterpene aldehydes: geranial (trans-isomer, E-citral or citral A) and neral (cis-isomer, Z-citral or citral B) [24] (see Supplementary Materials, pages S2–S4). The results showed that citral (**1**) exhibited potent activity against *L. amazonensis* and *T. cruzi*, displaying IC_50_ values of 10.9 and 24.8 μg/mL, respectively (Figure 2 and Table 2). In contrast, fraction EO1 presented a considerable challenge for purification due to its high chemical complexity and low polarity, which rendered conventional chromatographic techniques ineffective for isolating individual components. To overcome this limitation, GC-MS was employed as an alternative analytical approach to explore its chemical profile (Table 3 and Figure 3). A total of 22 compounds were identified based on these studies, with α-curcumene (57.7%), limonene-1,2-diol (26.8%), carveol (18.1%), 4-(4-methylphenyl)-pentanal (17.5%), and lavandulol acetate (16.9%) being the most abundant.

Our findings corroborate existing evidence of citral’s leishmanicidal activity, demonstrating its capacity to inhibit *Leishmania* parasite growth and proliferation, thus, reinforcing its potential as a hit compound against leishmaniasis. This aligns with prior studies that highlight plant-derived compounds as viable sources for antiparasitic drug development. Notably, while previous research has documented citral’s anti-leishmanial effects in essential oils or synthetic preparations, this study is the first to isolate and identify citral as the bioactive metabolite directly responsible for the observed activity within a specific plant extract, achieved through activity-guided fractionation [25,26,27].

Moreover, citral’s trypanocidal potential remains underexplored, with only a single published study examining its efficacy within an essential oil matrix against *Trypanosoma* species. The disparity between citral’s established leishmanicidal properties and its nascent investigation in trypanosomiasis models emphasizes the need for targeted research to elucidate its broader antiparasitic potential and refine its application across neglected tropical diseases [27].

### 2.2. Chemistry

**Figure 4:**
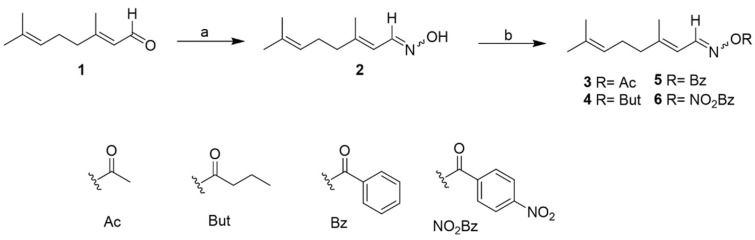
Synthesis of compounds **2–6**. (a) NH_2_OH.HCl, K_2_CO_3_, and MeOH. (b) Acetic anhydride/butyric anhydride/benzoyl chloride/4-nitrobenzoyl chloride, Et_3_N, and DMAP in acetone.

**Figure 5:**
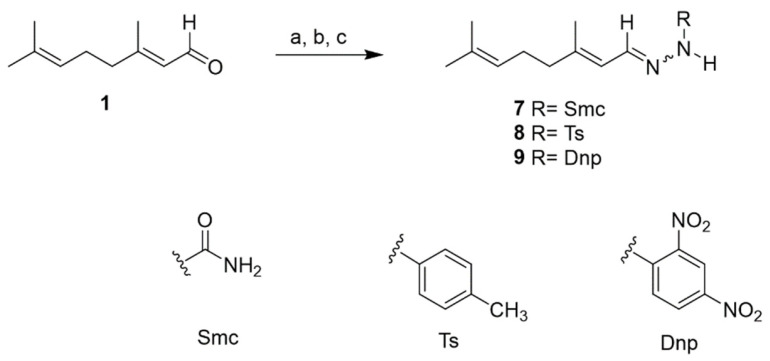
Synthesis of compounds **7**–**9**. (a) Semicarbazide hydrochloride, K_2_CO_3_, and MeOH; (b) 4-Toluenesulfonyl hydrazide and acetonitrile; and (c) 2,4-Dinitrophenylhydrazine, acetic acid, and MeOH.

Chemical modification of natural products remains an attractive strategy for developing new bioactive agents in medicinal chemistry [28]. Following the initial identification of the bioactive compound citral (**1**) through a bioassay-guided fractionation method, the next step of this work shifted to initiating a hit-to-lead optimization process. This effort focused on designing citral-containing Schiff bases to enhance their antiparasitic potency. In this study, the synthesis, structural characterization, and biological evaluation of eight compounds (**2**–**9**) were conducted. The synthetic approach used for this hit-to-lead optimization is comprehensively outlined in Figure 4 and Figure 5.

Initially, citral (**1**) underwent condensation reactions with diverse amines to produce Schiff-base compounds (**2** and **7**–**9**). The oxime (**2**) was then subjected to esterification with various anhydrides or acid chlorides, yielding the corresponding ester (**3**–**6**). In line with the spectroscopic characterization of the synthesized compounds, it is important to note the geometric nature of the starting material. Citral (a mixture of geranial and neral) and its derivatives exist as E/Z isomeric mixtures, as evidenced by duplicated signals in the NMR spectra. These spectroscopic features are consistent with literature data and are documented in the Supplementary Materials. All target compounds were purified using silica gel column chromatography, and their structural elucidation was achieved through comprehensive spectroscopic analysis and a comparison with reported data, as detailed in the Supplementary Materials (See S5–S13): citral oxime (**2**) [29], citral oxime acetate (**3**) [30], citral oxime butyrate (**4**) [30], citral oxime benzoate (**5**) [30], citral oxime 4-nitrobenzoate (**6**), citral semicarbazone **(7)** [29], citral 4-tosylhydrazone (**8**) [31], and citral 2,4-dinitrophenylhydrazone (**9**) [32]. Among these, compound **6** is reported here for the first time. Additionally, the spectroscopic data of compounds **3**–**5**, **8,** and **9** are provided herein, as they have not been previously reported.

### 2.3. In Vitro Kinetoplastid Activity of Citral Derivatives

Citral (**1**) and compounds **2–9** were assayed for their in vitro kinetoplastid activity against *L. amazonensis* promastigote and *T. cruzi* epimastigote stages. Furthermore, they were screened for their safety profile on the murine macrophage cell line J774.A1 for comparative purposes. Miltefosine and benznidazole, the reference drugs for treating leishmaniasis and Chagas disease, respectively, were also evaluated. Miltefosine exhibited an IC_50_ of 2.64 μM against *L. amazonensis*, while benznidazole showed an IC_50_ of 1.81 μM against *T. cruzi* (Table 4).

The results of the anti-kinetoplastid assays revealed that all citral-derived compounds exhibited no activity against *T. cruzi*. This suggests that structural alterations to the citral backbone may disrupt functional groups essential for antiparasitic efficacy, or that the parent compound’s activity is inherently limited against this protozoan. In contrast, the leishmanicidal activity analysis showed that all citral derivatives, except compounds **3** and **4** (IC_50_ ˃ 100 µM), which were inactive, displayed greater potency than the parent citral (8.47 μM), as indicated by their lower IC_50_ values, ranging from 10.62 to 43.75 µM. Among them, compound 9 emerged as the most active, exhibiting the lowest IC_50_ value. Nevertheless, all compounds remained less effective than miltefosine, the reference drug used as a positive control in this study. These results suggest that specific structural modifications of citral can enhance anti-kinetoplastid activity, although further optimization is required to achieve efficacy comparable to clinically used agents. Moreover, all active compounds exhibited good selectivity index (SI) values, with values ranging from 2.98 to 12.61. They were categorized according to the safety criteria described by Suffens [33], in which SI values of two or more are considered indicative of good selectivity.

### 2.4. In Vitro Leishmanicidal Activity on Intracellular Amastigotes

Based on the in vitro results against *L. amazonensis* promastigotes, compounds **2**, **8**, and **9** were selected for further evaluation against the intracellular amastigotes of *L. amazonensis* (Table 4). Their leishmanicidal activity demonstrated potent effects, with IC_50_ values ranging from 21.45 to 56.86 μM. However, their activity was slightly lower compared to miltefosine (IC_50_ value 3.12 μM). Additionally, compound **9** demonstrated low selectivity, as indicated by its CC_50_ value of 31.62 μM and a selectivity index (SI) value of 1.47. Based on its in vitro leishmanicidal activity on the promastigotes and amastigotes stages of *L. amazonensis*, compound **9** was selected for further study to elucidate its mechanism of action.

### 2.5. Mechanism of Action of Compound **9**

**Figure 6:**
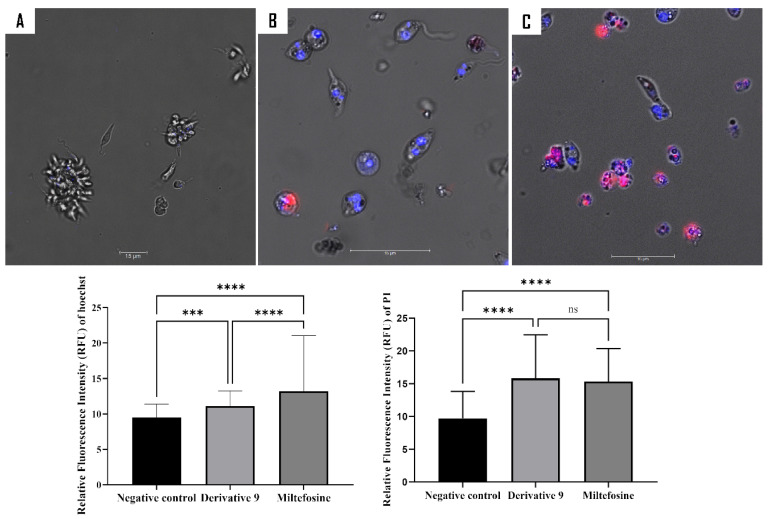
Determination of the type of cell death using Hoechst/PI staining after 24 h of incubation with IC_90_ against *L. amazonensis* promastigotes. The images (63×), obtained using a Leica SPE confocal microscopy, are representative of the observed cell population. The histogram illustrates the distribution of fluorescence intensities for each sample, as obtained by the EVOS M5000 software (version 1.5.1500.493). The differences between the values were assessed using one-way analysis of variance (ANOVA). In the context of statistical analysis, the following symbols are used to denote the level of significance of the results obtained: *** *p* < 0.001; **** *p* < 0.0001; and ns: non-significance. (**A**) Negative control, (**B**) compound **9**, and (**C**) miltefosine.

To further investigate the action mechanism of compound **9** on *L. amazonensis*, a series of fluorescence assays was conducted. A double staining assay was employed to ascertain the type of cell death induced by compound **9** on *L. amazonensis*. After a 24 h period of incubation with the relevant IC_90_ of the compound, the resulting fluorescence was measured in both the treated and untreated cells using an image-based cytometer, the EVOS M5000. The results obtained are presented as histograms, which are an effective method of depicting differences in fluorescence distribution between treated and untreated cells (Figure 6).

**Figure 7:**
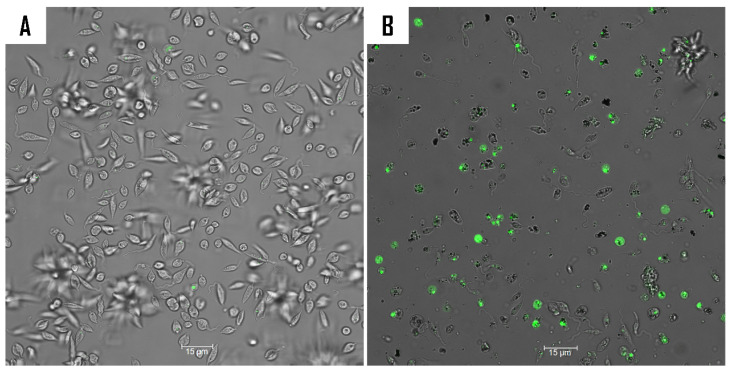

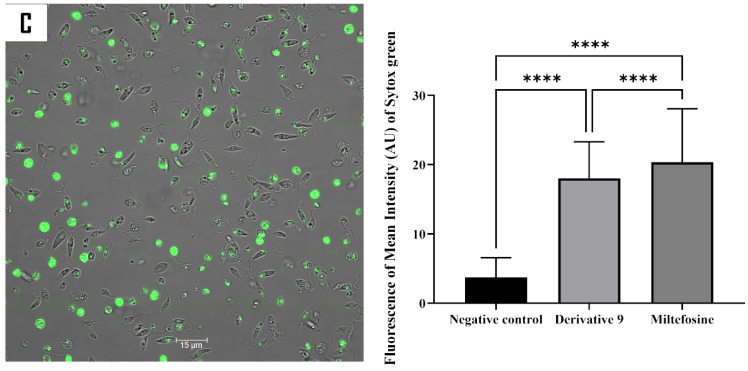
Analysis of plasma membrane permeability in *L. amazonensis* using SYTOX^®^ Green staining after 24 h of incubation with compound **9** (**B**) and miltefosine (**C**). The images (63×) are representative of the cell population observed in the performed experiments. Image acquisition was facilitated by a Leica SPE confocal microscope. The histogram illustrates the distribution of fluorescence intensities for each sample, as obtained by the EVOS M5000 software. The differences between the values were assessed using one-way analysis of variance (ANOVA). **** *p* < 0.0001. (**A**) Negative control.

SYTOX^®^ Green, a DNA-specific stain, was used in this study to examine the impact of compound **9** on cell membrane permeability (Figure 7). The fluorochrome enters cells with compromised membrane permeability and emit green fluorescence upon binding to DNA [34]. Following a 24 h treatment period, the fluorescence intensity was measured using the EVOS M5000. The resulting fluorescence intensities are presented in a histogram. Although, both treatments can affect the cell’s membrane permeability. Miltefosine resulted in a greater degree of damage to membrane permeability compared to compound **9**.

A series of experiments was conducted to examine the impact of the tested compound on the function of the mitochondria. This involved three different assays. First, the assessment of mitochondrial membrane potential was conducted using the JC-1 dye. Second, the measurement of ATP levels within the cell was taken. Finally, the intracellular reactive oxygen species levels were determined. The mitochondria constitute the principal organelle responsible for producing intracellular energy and determining the type of cell death that occurs in the cells. JC-1 staining was used to differentiate between healthy and damaged mitochondria.

**Figure 8:**
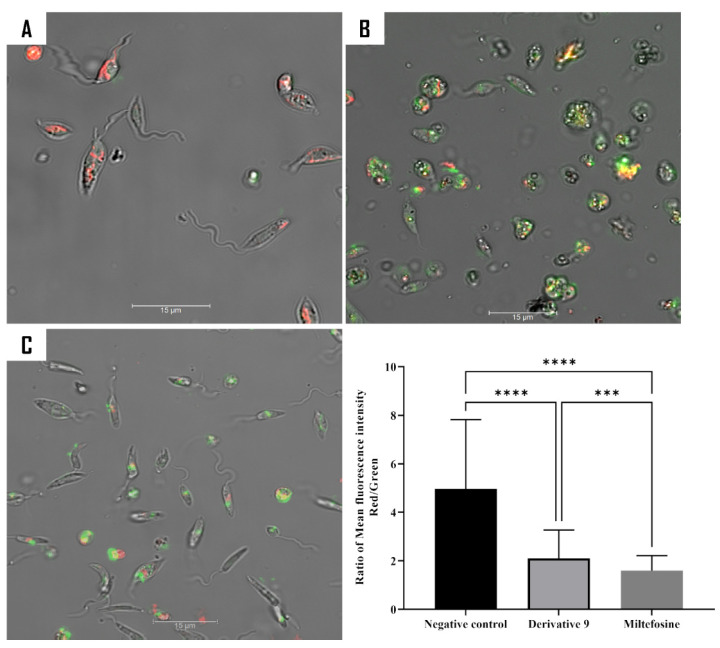
The mitochondrial membrane potential in *L. amazonensis* promastigotes was evaluated using JC-1 staining following a 24 h incubation period with compound **9** (**B**) and miltefosine (**C**). The images (63×), obtained by a Leica SPE confocal microscopy system, are representative of the cell population observed in the performed experiments. The histogram graphs represent the ratio of mean fluorescence obtained by the EVOS M5000. The differences between the values were assessed using one-way analysis of variance (ANOVA). *** *p* < 0.001; **** *p* < 0.0001. (**A**) Negative control.

Following a 24 h incubation period with compound **9**, an increase in green fluorescence was observed, which is indicative of the inability of JC-1 to form aggregates due to the low ∆Ψm. The mean fluorescence for both forms of JC-1 (aggregate and monomeric) was determined by means of the EVOS M5000 software, and the ratio of red fluorescence to green fluorescence was calculated. As demonstrated in the histogram (Figure 8), the potential of the mitochondrial membrane of *L. amazonensis* treated with the compound decreased by 50% and 75% in comparison with untreated cells, respectively, for compound **9** and miltefosine. As a consequence of the collapse of the mitochondrial membrane potential, the ATP production process can be inhibited. In the assay, a luminescence-based method was used to quantify the level of ATP produced by treated and untreated cells. As expected, compound **9** and miltefosine led to a significant decrease in ATP production, with 46.35% and 74.11% reductions in *L. amazonensis* in comparison to the negative controls.

**Figure 9:**
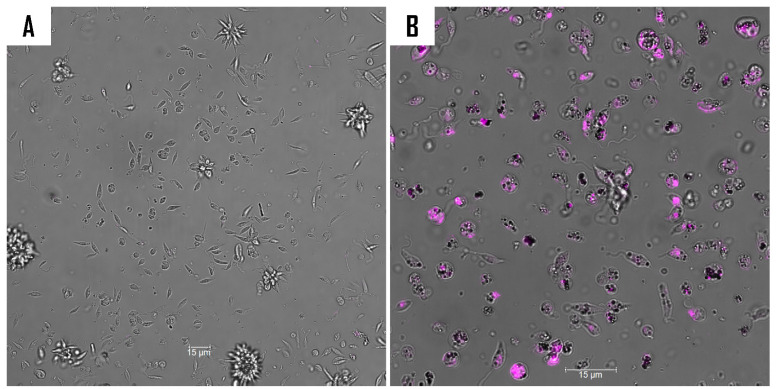

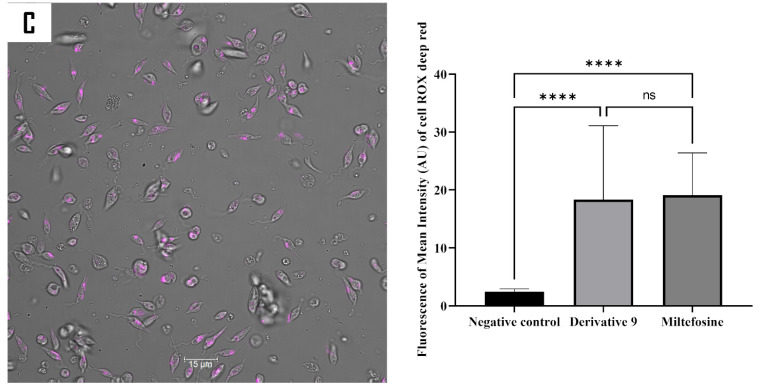
Analysis of ROS production in *L. amazonensis* by compound **9** (**B**) and miltefosine (**C**). The images (63×) are representative of the cell population observed in the performed experiments. The histogram was obtained using an EVOS M5000 Cell Imaging System, Life Technologies, Spain. ns: Non-significant; **** *p* < 0.0001. (**A**) Negative control.

Damage to mitochondrial functionality would consequently lead to an imbalance between free radicals and antioxidant levels, thereby resulting in the disturbance of the cell’s redox state. The levels of intracellular reactive oxygen species (ROS) were measured after 24 h of incubation using CellROX Deep Red^TM^. The treatment resulted in a significant increase in emitted fluorescence, indicating that compound **9** can induce oxidative stress in *L. amazonensis* (Figure 9).

## 3. Materials and Methods

### 3.1. General Procedures

Bruker Avance 500 and 600 spectrometers were employed for the acquisition of 1D NMR spectra (Bruker, Wissembourg, France). Chemical shifts were reported in δ (ppm), while coupling constants were expressed in Hz, with reference to the residual deuterated solvent (CDCl_3_: δ_H_ 7.26 and δ_C_ 77.16), and TMS was utilized as an internal reference. ESIMS and HRESIMS (positive mode) analyses were conducted using an LCT Premier XE Micromass Autospec spectrometer (Waters, Barcelona, Spain). Column chromatography was performed using silica gel 60 (particle sizes 15–40 and 63–200 µm), analytical, preparative and high-performance thin layer chromatography (TLC) were used, along with Polygram Sil G/UV254 plates (Panreac, Barcelona, Spain). High-performance TLC used HPTLC-Platten Nano-Sil 20 UV_250_ plates (Panreac, Barcelona, Spain). The progress of all reactions was monitored using TLC, and the visualization of spots was achieved through UV light exposure and the heating of silica gel plates sprayed with H_2_O-H_2_SO_4_-AcOH (1:4:20). Unless specified otherwise, solvents and reagents were procured from commercial suppliers and used without further purification. Analytical-grade solvents from Panreac and reagents from Sigma Aldrich (St. Louis, MO, USA) were employed. Citral (**1**), used as the starting material, was sourced from Cymit Química S. L. (Barcelona, Spain).

### 3.2. Plant MaterialCitral

*Aloysia citrodora* Paláu leaves were from Jendouba, a north-western region of Tunisia, in August 2021. Botanical identification was carried out by Dr. Fatma Tajini, Associate Professor at the Higher Institute of Biotechnology of Béja, University of Jendouba. A voucher specimen (ISBB-AC-2021-08) was deposited in the Herbarium Higher Institute of Biotechnology of Béja, Tunisia.

### 3.3. Preparation of Aloysia citrodora Essential Oil

The air-dried leaves from *A. citrodora* (7.0 kg) were extracted by steam distillation with water (8 L) and heating the mixture at 90 °C for 3 h, yielding 11.1 g of essential oil (EO). The EO was stored in a dark container at 4 °C until it was used. An aliquot (30.0 mg) of EO was assayed on the pathogenic parasites *L. amazonensis* and *T. cruzi*, exhibiting remarkable kinetoplastid activity.

### 3.4. Gas Chromatography–Mass Spectrometry Analysis

An 8890 gas chromatograph (Agilent Technologies, Santa Clara, CA, USA) coupled to a 5977B single quadrupole mass spectrometer (Agilent Technologies) was used for the identification of the main constituents of the essential oil. For the analysis of the EO1 fraction, an 8890 GC (Agilent Technologies) coupled to a 7000D triple quadrupole mass spectrometer (Agilent Technologies) was used. In both cases, the same experimental conditions were employed. An HP-5 MS capillary column (30 m L. × 0.25 mm I.D. and 0.25 µm film thickness) was used. Helium was employed as the carrier gas at a constant flow rate of 1.0 mL·min^−1^. The inlet was kept at 250 °C; the injection volume was 1 µL, and the injection mode was *split-less*. The oven temperature gradient started at 40 °C. This temperature was held for 1 min. After that, the temperature increased at 4 °C·min^−1^ up to 300 °C, and finally this temperature was held for 4 min. The transfer line was kept at 280 °C. In the MS, the temperatures of the ion source and quadrupoles were 230 °C and 150 °C, respectively. The ion source operated in electron impact (EI) mode at 70 eV. The MS operated in scan mode, with a solvent delay of 5 min, a scan interval for mass spectral data acquisition of 1.0 s, and data acquisition over a range mass from 40 to 400 *m*/*z*.

For analysis, the essential oil and the fraction EO1 were diluted in cyclohexane and injected in GC-MS in triplicate. Identification was assessed by comparing the spectral data with the spectra of the NIST v.2014 library and through both the Agilent Masshunter Workstation Qualitative Analysis (version 10) and Agilent Masshunter Unknowns Analysis (version 10.1) software. Only the compounds identified in the three chromatograms at the same retention time and with an R Match value higher than 750 were considered. The identification was then verified by the determination of the Kovats Indexes (KIs). The experimental KI (KI_exp_) was obtained based on the retention of a series of linear alkanes (C_4_–C_37_) injected at the same chromatographic conditions. These values were compared with reference KI values (KI_theo_) from the literature.

### 3.5. Bioactivity-Guided Chromatography Fractionation and Isolation

The *A. citrodora* essential oil (2.0 g) was subjected to further fractionation by silica gel column chromatography (CC), using pentane (200 mL) and mixtures of hexane–EtOAc (200 mL) of increasing polarity (10:0 to 0:10) as eluent to afford thirteen fractions, which were combined based on their TLC profile in five fractions (EO1–EO5).

Kinetoplastid activity revealed that the sub-fractions EO1 and EO2 were the most active against the tested parasites. EO2 was subjected to a chromatography step until the pure metabolites were obtained (Figure 1). In fact, using an isocratic mixture of hexane–EtOAc (9:1.5), an aliquot of the EO2 fraction (30.0 mg) was fractionated by preparative TLC to give four sub-fractions (EO2A-EO2D). Using an isocratic mixture of hexane–EtOAc (9:1.5), the sub-fraction EO2C (4.2 mg) was purified by high-performance TLC to yield the pure compound (3.4 mg), which was identified as citral (**1**). Structural characterization was carried out by the analysis of NMR spectroscopic data (Supplementary Materials, page S5) and a comparison with data reported in the literature [24].

### 3.6. Compounds Synthesis

The compounds (**2–9**) were synthesized following a standard protocol (Supplementary Materials, pages S2–S4).

### 3.7. Biological Assays

#### 3.7.1. In Vitro Leishmanicidal Activity on *L. amazonensis* Promastigote

The assay was conducted using the Alamar Blue^®^ method as previously described [35]. Briefly, the promastigotes of *L. amazonensis* were grown at 26 °C in Schneider’s medium (US Biological, Life sciences, Salem, MA, USA) and supplemented with 10% heat-inactivated fetal bovine serum and 0.04% of sodium bicarbonate. In 96-well microtiter plates (Corning^TM^, Corning, NY, USA), serial dilutions of the citral derivative were first prepared, and later 10^6^ parasites/mL were added into each well. Subsequently, 10% Alamar Blue^®^ was added to the entire plate. The plates were incubated for 72 h at 26 °C.

#### 3.7.2. In Vitro Trypanocidal Activity on *T. cruzi* Epimastigotes

In a 96-well plate, a serial dilution of the tested compounds was performed in the liver infusion tryptose medium supplemented with 10% heat-inactivated fetal bovine serum. Later, 100 µL of 5 × 10^5^ cells/mL of epimastigotes was added to each well. Afterwards, 20 µL of alamarBlue^®^ was added to each well, and the plate was maintained at 26 °C for 72 h [32].

#### 3.7.3. In Vitro Leishmanicidal Activity on *L. amazonensis* Intracellular Amastigotes

The most active compounds against the promastigotes of *Leishmania amazonensis* was evaluated against the intra-macrophagic stage of *Leishmania amazonensis* as previously described [36,37].

#### 3.7.4. Cytotoxicity Activity on Murine Macrophages

The cytotoxicity effect of the tested compounds was conducted using macrophages of the murine cell line J774.A1 (American Type Culture Collection #TIB-67) as previously described [36]. The cells were maintained in the DMEM medium at 37 °C in a 5% CO_2_ humidified incubator. All the plates containing alamarBlue^®^ were analyzed using the plate reader EnSpire Multimode Plate Reader^®^ at excitation and emission wavelengths of 570 and 585 nm, respectively (PerkinElmer, ThermoFisher Scientific, Madrid, Spain). The IC_50_ (concentrations able to inhibit 50% of parasites), IC_90_ (concentrations able to inhibit 90% of parasites), and CC_50_ (concentration able to inhibit 50% of murine macrophages) values were determined for each compound by nonlinear regression using the GraphPad Prism 9 software.

### 3.8. Elucidation of the Mechanism of Action

The effect of compound **9** to modulate cell features was studied in the *L. amazonensis* parasites using various specific commercial kits. Parasite cells were incubated with the compound at the previously calculated IC_90_ for 24 h. Subsequently, cells were washed and submitted to different treatments as mentioned by the manufacturer’s instructions. In all the assays, the plate reader EnSpire Multimode Plate Reader^®^ (PerkinElmer, ThermoFisher Scientific, Madrid, Spain) and/or the image base system fluorescence microscope EVOS M5000 (version 1.5.1500.493) were used for fluorescence intensity quantification. For each protocol, images of representative population cells were obtained using the inverted confocal microscope Leica DMI 4000 B with the Leica Application Suite X 3.5.5.19976 software and Leica HC PL APO 63x/1.40 OIL CS2 Objective (Barcelona, Spain).

#### 3.8.1. Double Stain Assay for Detecting Programmed Cell Death (PCD)

To identify the type of cell death occurring in treated *Leishmania amazonensis*, the Hoechst 33342 and propidium iodide (PI) dyes were employed. Although both dyes are DNA-affine, Hoechst 33342 is cell-permeable and is known to distinguish apoptotic cells from healthy or necrotic cells [34]. In contrast, PI is cell-impermeable and stains only dead cells, such as those in late-stage apoptosis or necrosis. Post-treatment, three distinct staining patterns could be observed: blue fluorescence indicating cells undergoing apoptosis; violet coloration resulting from the merge of blue and red fluorescence, representing late apoptotic cells; and red fluorescence corresponding to dead cells.

#### 3.8.2. Analysis of Plasma Membrane Permeability

SYTOX^TM^ Green (Life Technologies, Madrid, Spain), a cell-impermeable DNA-binding stain, was used to evaluate membrane permeability and integrity. The assay was conducted as detailed in the manufacture instructions and as previously described [38].

#### 3.8.3. Analysis of Mitochondrial Function

To evaluate the damage caused by the tested compound on mitochondrial function, mitochondrial membrane potential, the adenosine triphosphate (ATP) level, and reactive oxygen species (ROS) were measured as follows: The evaluation of the mitochondrial membrane potential was carried out using the JC-1 assay. JC-1 is a specific stain of the mitochondria which fluorescence emission depends on the mitochondrial membrane potential. In healthy mitochondria, JC-1 agglomerates and emits red fluorescence, while unhealthy ones remain as a monomer and emit green fluorescence. Thus, mitochondria with collapsed membrane potential would exhibit a low red/green fluorescence intensity ratio. The assay was performed according to the manufacture instructions as detailed in previous work [38].

The adenosine triphosphate (ATP) level was measured using the CellTiter-Glo Luminescent Cell Viability Assay (Promega, Madrid, Spain) [38]. The mitochondrion in which most cellular oxidations occur is considered the main source of cell ATP, and any damage and/or malfunction of this organelle generate the depletion of ATP levels.

The measure of reactive oxygen species (ROS) was measured after treatment for 24 h using the CellROX^®^ Deep Red fluorescent probe (Invitrogen, Madrid, Spain) [38]. The mitochondrion constitute an essential organelle to neutralize the reactive oxygen species; the malfunction of this organelle would generate an imbalance between the antioxidants and the reactive oxygen.

### 3.9. Statistical Analysis

The percentage of inhibition and 50% inhibitory concentration (IC_50_) was determined by linear regression analysis with 95% confidence limits. All experiments were performed three times in duplicate, and the mean values were also calculated. A paired two-tailed *t*-test was used for the analysis of the data. Values of *p* < 0.05 were considered significant. The statistical analysis of the inhibition curves was undertaken using the Sigma Plot 12.0 software program (Systat Software Inc., San Jose, CA, USA). All antiprotozoal tests were performed in triplicate, and the results are presented as average values ± the standard deviation. Differences between the values were assessed using one-way analysis of variance (ANOVA), where *** *p* < 0.001; **** *p* < 0.0001; and ns: non-significance.

## 4. Conclusions

Natural products continue to serve as an invaluable reservoir of molecular diversity, consistently driving the discovery and development of novel therapeutic agents. In this study, citral was successfully isolated from the essential oil of *A. citrodora* leaves through bioassay-guided fractionation and identified as an active anti-kinetoplastid compound against *L. amazonensis* and *T. cruzi*. Building upon this finding, a series of citral-derived Schiff bases was synthesized and evaluated for their antiparasitic activity. While the chemo-modulation of citral resulted in compounds that were inactive against *T. cruzi*, a contrasting trend was observed in the leishmanicidal assay, where nearly all compounds exhibited enhanced potency compared to the parent molecule. Among them, compound **9** stood out as the most active compound, showing the lowest IC_50_ value within the series. However, despite the improved activity relative to citral, all compounds remained less potent than miltefosine, which was employed as the positive control. Further mechanistic studies revealed that the antiparasitic effect of compound **9** is mediated through the induction of mitochondrial-dependent apoptosis in the parasite.

This study highlights the potential of *Aloysia citrodora* as a valuable source of lead compounds for the development of novel leishmanicidal agents. These findings further underscore the enduring relevance of natural products and their semi-synthetic compounds in the ongoing pursuit of effective antiparasitic therapies.

## Figures and Tables

**Table 1:** Chemical composition of *A. citrodora* essential oil by GC/MS.

N°	Compound ^a^	Composition ^b^ (%)	Formula	RT ^c^ (min)	KI_exp_^d^	KI_theo_^e^
**1**	Sabinene	2.59	C_10_H_16_	9.44	969	974
**2**	Sulcatone	1.85	C_8_H_14_O	9.95	-	-
**3**	D-Limonene	16.0	C_10_H_16_	11.4	1089	1018
**4**	Eucalyptol	6.91	C_10_H_18_O	11.5	1093	1059
**5**	α-Terpineol	1.92	C_10_H_18_O	17.2	1387	1190
**6**	Neral	11.1	C_10_H_16_O	19.0	1377	1228
**7**	α-Citral	11.8	C_10_H_16_O	20.0	1413	1174
**8**	Nerolidyl acetate	2.08	C_17_H_28_O	23.7	1701	1805
**9**	Caryophyllene	2.87	C_15_H_24_	24.8	1756	1423
**10**	α-Curcumene	8.71	C_15_H_22_	26.8	1586	1524
**11**	Spathulenol	4.21	C_15_H_24_O	29.6	1658	1576
**12**	Caryophyllene oxide	6.34	C_15_H_24_O	29.7	1663	1581

^a^ Tentative identification. ^b^ Percentage of chromatographic peak area, estimated from the ratio of peak area of each identified compound and the total peak area of all peaks of the chromatograms (considering both identified and non-identified compounds). ^c^ Retention time. ^d^ Experimental Kovats index. ^e^ Theoretical Kovats index.

**Table 2:** Anti-kinetoplastid activity against *L. amazonensis* promastigotes and *T. cruzi* epimastigotes of the essential oil (EO), fractions (EO1-EO5), subfractions (EO2A-EO2D) and isolated compound **1** (citral) from *A. citrodora* leaves.

Samples	*L. amazonensis* IC_50_ µg/mL ^a^	*T. cruzi* IC_50_ µg/mL	Samples	*L. amazonensis* IC_50_ µg/mL	*T. cruzi* IC_50_ µg/mL
EO	10.67 ± 2.42	22.60 ± 7.84	EO2A	˃100	˃100
EO1	17.38 ± 5.77	24.35 ± 4.82	EO2B	˃100	˃100
EO2	36.71 ± 4.07	59.11 ± 4.97	EO2C	12.62 ± 2.30	26.27 ± 2.07
EO3	˃100	˃100	EO2D	61.91 ± 6.32	34.41 ± 4.86
EO4	˃100	˃100	Citral (1)	8.47 ± 4.59	12.90 ± 1.92
EO5	˃100	˃100	Miltefosine ^b^	2.64 ± 0.09	
			Benznidazole ^c^		1.81 ± 0.50

^a^ IC_50_: Concentrations able to inhibit 50% of the growth of the tested parasite, expressed in µg/mL ± the standard deviation (SD). ^b^ Miltefosine was used as a positive control against *L. amazonensis*. ^c^ Benznidazole was used as the positive controls against *T. cruzi*. Anti-kinetoplastid activity was performed as independent experiments in triplicate.

**Table 3:** Chemical composition of fraction EO1 by GC-MS.

N°	Compound ^a^	Composition ^b^ (%)	Formula	RT ^c^ (min)	KI_exp_^d^	KI_theo_^e^
**1**	Eucalyptol	10.6	C_10_H_18_O	11.9	1030	1032
**2**	Carveol	18.1	C_10_H_16_O	18.6	1220	1219
**3**	D-Verbenone	3.31	C_10_H_14_O	18.9	1231	1324
**4**	Citral	1.13	C_10_H_16_O	20.3	1272	1276
**5**	Myrtenyl acetate	4.49	C_12_H_18_O_2_	20.8	1287	1327
**6**	Acetomesitylene	2.29	C_9_H_12_	21.7	1312	1369
**7**	Limonene-1,2-diol	26.8	C_10_H_18_O_2_	22.7	1344	1321
**8**	2-(Phenylcyclobutyl) benzene	0.99	C_16_H_16_	23.0	1354	1749
**9**	Lavandulol acetate	16.9	C_12_H_20_O_2_	24.1	1386	1270
**10**	4-(4-Methylphenyl) pentanal	17.5	C_12_H_16_O	24.7	1404	1429
**11**	2-epi-α-Funebrene	3.08	C_15_H_24_	25.0	1413	1403
**12**	β-Copaene	5.25	C_15_H_24_	25.2	1422	1432
**13**	Neryl (S)-2-methylbutanoate	7.33	C_15_H_26_O_2_	26.9	1477	1576
**14**	Isoledene	2.03	C_15_H_24_	27.0	1478	1375
**15**	α-Curcumene	57.7	C_15_H_22_	27.2	1465	1483
**16**	(±)-γ-Muurolene	3.89	C_15_H_24_	28.1	1539	1477
**17**	cis-Calamenene	2.11	C_15_H_22_O	28.4	1561	1531
**18**	β-Calacorene	1.00	C_15_H_22_	28.9	1601	1563
**19**	α-Calacorene	6.62	C_15_H_22_	28.9	1601	1542
**20**	Caryophyllene oxide	10.7	C_15_H_24_O	29.2	1624	1581
**21**	Norbourbonone	3.21	C_9_H_12_O	29.5	1639	1563
**22**	α-Dehydro-ar-himachalene	1.61	C_15_H_22_	29.9	1663	1601

^a^ Tentative identification. ^b^ Percentage of chromatographic peak area. ^c^ Retention time. ^d^ Experimental Kovats index. ^e^ Theoretical Kovats index.

**Table 4:** Kinetoplastid activity of citral (**1**) and compounds (**2–9**) on the promastigotes and amastigotes of *L. amazonensis* and cytotoxicity against murine macrophage cells.

Cmpds	*L. amazonensis* Promastigotes	Selectivity	*L. amazonensis* Amastigotes	Selectivity	Cytotoxicity
	IC_50_^a^	SI ^c^	IC_50_^a^	SI ^c^	CC_50_^b^
**1**	55.64 ± 30.02	6.97			>300
**2**	40.84 ± 19.43	12.61	56.86 ± 19.13	9.39	>300
**5**	29.30 ± 8.07	10.41			>300
**6**	34.17 ± 14.44	9.25			>300
**7**	43.75 ± 12.05	9.29			>300
**8**	40.17 ± 22.43	7.78	34.45 ± 6.56	9.07	>300
**9**	10.62 ± 4.78	2.98	21.45 ± 0.65	1.47	31.62 ± 1.96
**M** ^d^	6.47 ± 0.22	11.16	3.12 ± 0.30	23.13	72.18 ± 8.85

^a^ IC_50_: Concentrations able to inhibit 50% of parasites, expressed as µM ± the standard deviation (SD). ^b^ CC_50_: Concentration able to inhibit 50% of murine macrophages, expressed as µM ± the standard deviation (SD). ^c^ SI: Selectivity index (CC_50_/IC_50_).^d^ M: Miltefosine was used as a positive control against *L. amazonensis*. Anti-kinetoplastid activity and cytotoxicity assays were performed as independent experiments in triplicate. Compounds **3** and **4** were excluded due to their low antiparasitic activity.

## Data Availability

Data are contained within the article and Supplementary Materials.

## Supplementary Materials

The supporting information can be downloaded at https://www.mdpi.com/article/10.3390/ijms26125697/s1.
