# Supplementary material for: Bioprospecting for Anti-Kinetoplastid Drug Discovery from Aloysia citrodora Essential Oil
Source: Int J Mol Sci. 2025 Jun 13;26(12):5697. doi: 10.3390/ijms26125697 (PMC12193109; doi:10.3390/ijms26125697)
Supplement: Supplementary file 1 [file ijms-26-05697-s001.zip › ijms-3663080-supplementary.pdf]

Bioprospecting for Anti-kinetoplastid Drug Discovery from *Aloysia citrodora* Essential Oil

Amani Omrani[1,2,3], Meriam Ben Youssef[1,2,3], Ines Sifaoui [1,4,5], Eduardo Hernández-Álvarez[3], María J. Trujillo-Rodríguez[1,6], Montse Saura-Cayuela[1,6], Verónica Pino[1,5,6], Hichem Sebai[2], Isabel L. Bazzocchi[3], Jacob Lorenzo-Morales[1,4,5], José E. Piñero [1,4,5], Ignacio A. Jiménez[3,*]

[1] Instituto Universitario de Enfermedades Tropicales y Salud Pública de Canarias, Universidad de La Laguna, 38296 San Cristóbal de La Laguna, Santa Cruz de Tenerife, Spain
[2] Laboratory of Functional Physiology and Valorization of Bio-Ressources, Higher Institute of Biotechnology of Beja, University of Jendouba, Beja 382-9000, Tunisia
[3] Instituto Universitario de Bio-Orgánica Antonio González and Departamento de Química Orgánica, Universidad de La Laguna, Avenida Astrofísico Francisco Sánchez 2, 38206 La Laguna, Tenerife, Spain
[4] Departamento de Obstetricia y Ginecología, Pediatría, Medicina Preventiva y Salud Pública, Toxicología, Medicina Legal y Forense y Parasitología, Universidad de La Laguna, C/ Sta. María Soledad s/n, 38200 San Cristóbal de La Laguna, Santa Cruz de Tenerife, Spain
[5] Consorcio Centro de investigación Biomédica en Red, Área de Enfermedades Infecciosas, Instituto de Salud Carlos III, Av. Monforte de Lemos 3-5, Pabellón 11, 28029 Madrid, Spain
[6] Departamento de Química, Unidad Departamental de Química Analítica, Universidad de La Laguna (ULL), 38206 San Cristóbal de La Laguna, España

Table of contents

Pages S2-S4: Experimental part. Synthesis of compounds **2-9**.

Page S5: **Figures S1 and S2**, ^{1}H NMR and ^{13}C NMR spectra of compound **1**

Page S6: **Figures S3, S4 and S5**, ^{1}H NMR,^{13}C NMR and Mass spectra of compound **2**

Page S7: **Figures S6, S7 and S8**, ^{1}H NMR, ^{13}C NMR and Mass spectra of compound **3**

Page S8: **Figures S9, S10 and S11**, ^{1}H NMR, ^{13}C NMR and Mass spectra of compound **4**

Page S9: **Figures S12, S13 and S14**, ^{1}H NMR, ^{13}C NMR and Mass spectra of compound **5**

Page S10: **Figures S15, S16 and S17**, ^{1}H NMR, ^{13}C NMR and Mass spectra of compound **6**

Page S11: **Figures S18, S19 and S20**, ^{1}H NMR, ^{13}C NMR and Mass spectra of compound **7**

Page S12: **Figures S21, S22 and S23**, ^{1}H NMR, ^{13}C NMR and Mass spectra of compound **8**

Page S13: **Figures S24, S25 and S26**, ^{1}H NMR, ^{13}C NMR and Mass spectra of compound **9**

The compound (**2-9**) were synthesized following a standard protocol. All compounds are previously known documented in the literature, with the exception of compound **6**, which is being reported for the first time in this study.

*3.6.1. Preparation of compound **2**.*

A solution of citral (53.3 mg, 0.35 mmol) in methanol (2 mL) was added to a stirred solution of hydroxylamine hydrochloride (210.8 mg, 3.0 mmol) and potassium carbonate (420.8 mg, 3.0 mmol) in methanol (4 mL). The resulting mixture was stirred at 22 ºC for 30 min and the progress was monitored by TLC, using mixture of hexanes-EtOAc (8:2). Once the reaction has concluded, the methanol was removed. Water (10 mL) was added to the residue and then was extracted by liquid-liquid partition using diethyl ether (3 x 10 mL). The combined organic phases were dried over magnesium sulphate, filtering off the drying agent by gravity. The solvent was removed under reduced pressure to give the desired compound **2** (29.4 mg, 50.2 %).

*3.6.2. General procedure of compound synthesis **3-6***

An excess of the corresponding carboxylic acid derivative was added to a solution of **2**, triethylamine (Et_3N), and catalytic amount of 4-dimethylaminopyridine (DMAP) in acetone. The resulting mixture was stirred at 22 °C for 30 min. The reaction was monitored by TLC using mixtures of hexane-diethyl ether (5:5). Once the reaction has been concluded, the solvent was removed under reduced pressure. The residue was purified by a silica gel column chromatography (CC) using a hexane-diethyl ether gradient with increasing polarity (from 7:3 to 5:5) to yield the corresponding esters (**3-6**).

*3.6.2.1. Preparation of compound **3**.*

Following the procedure as described for oxime acylation, acetic anhydride (43.5 mg, 0.4 mmol), compound **2** (10.0 mg, 0.06 mmol), Et_3N (0.1 mL) and DMAP (3.1 mg) in acetone (0.5 mL), provide the desired compound **3** (1.0 mg, 8.0 %). Citral-oxime acetate (**3**). ^{1}H NMR (500 MHz, acetone-d_6) δ 8.36 (1H, d, J = 10.5 Hz, H-1), 8.31 (1H, d, J = 10.5 Hz, H-1), 5.98 (1H, d, J = 10.5 Hz, H-2), 5.13 (1H, d, J = 6.8 Hz, H-6), 2.36 (1H, t, J = 7.5 Hz, H-5), 2.23 (2H, m, H-4), 2.19 (1H, t, J = 7.5 Hz, H-5), 1.95, 1.94 (3H, d, J = 1.2 Hz, H-9), 1.67, 1.65 (3H, s, H-10), 1.62, 1.60 (3H, s, H-8), acetate [2.09, 2.10 (3H, s)]; ^{13}C NMR (125 MHz, acetone-d_6) δ 154.8, 154.5 (CH-1), 145.8, 145.7 (C-3), 133.2, 132.7 (C-7), 124.2, 124.1 (CH-6), 118.5, 117.6 (CH-2), 40.8 (CH_2-4), 33.3 (CH_2-5), 27.4, 26.8 (CH_3-9), 25.8, 24.6 (CH_3-10), 17.8, 17.5 (CH_3-8), acetate [168.8 (-COO-),19.6 (CH_3),]; ESIMS *m/z* 232 $[M + Na]^+$ (100), HRESIMS *m/z* 232.1312 (calcd for $C_{12}H_{19}NO_2Na$, $[M+Na]^+$, 232.1313).

*3.6.2.2. Preparation of compound **4**.*

Following the procedure as described for oxime acylation, butyric anhydride (100.8 mg, 0.6 mmol), compound **2** (100.8 mg, 0.6 mmol), Et_3N (0.2 mL) and DMAP (6.9 mg) in diethyl ether (1 mL), provide the desired compound **4** (2.8 mg, 10.7 %). Citral-oxime butyrate (**4**). ^{1}H NMR (500 MHz, acetone-d_6) δ 8.37 (1H, d, J = 10.5 Hz, H-1), 8.32 (1H, d, J = 10.5 Hz, H-1), 5.99

(1H, d, J = 10.5 Hz, H-2), 5.13 (1H, t, J = 5.8 Hz, H-6), 2.36 (1H, t, J = 7.5 Hz, H-5), 2.23 (2H, m, H-4), 2.18 (1H, t, J = 7.5 Hz, H-5), 1.95 (3H, d, J = 1.2 Hz, H-9), 1.94 (3H, d, J = 1.2 Hz, H-9), 1.67, 1.65 (3H, s, H-10), 1.61, 1.60 (3H, s, H-8), butyrate [2.38 (2H, t, J = 7.7 Hz), 1.66 (2H, m), 0.95 (3H, t, J = 7.6 Hz)]; ^{13}C NMR (125 MHz, acetone-d_6) δ 154.9, 154.6 (CH-1), 154.4, 154.3 (C-3), 133.2, 132.7 (C-7), 124.2, 124.1 (CH-6), 118.6, 117.7 (CH-2), 40.8 (CH_2-4), 33.3 (CH_2-5), 27.4, 26.8 (CH_3-9), 25.8, 24.6 (CH_3-10), 18.9, 17.8 (CH_3-8), butyrate [171.2 (-COO-), 35.0 (CH_2), 25.8 (CH_2),13.8 (CH_3)]; ESIMS *m/z* 260 $[M+Na]^+$ (100), HRESIMS *m/z* 260.1631 (calcd for $C_{14}H_{23}NO_2Na$, $[M+Na]^+$, 260.1626).

3.6.2.3. Preparation of compound ***5****.*

The solution of **2** (10.1 mg, 0.06 mmol), triethylamine (0.1 mL), benzoyl chloride (12.1 mg, 0.09 mmol) and catalytic amount of DMAP (4.8 mg) in acetone (1.0 mL) was stirred for 30 min. The residue was purified by silica gel CC, yielding the impure compound 4. Potassium fluoride (60.4 mg) was added to the solution of impure compound in acetone (0.5 mL), and stirred at room temperature for 2 h, and then was filtrated through celite. The solvent was removed under reduced pressure to afford the corresponding compound **5** (4.7 mg, 28.9 %). Citral-oxime benzoate (**5**). ^{1}H NMR (500 MHz, acetone-d_6) δ 8.67 (1H, d, J = 10.5 Hz, H-1), 8.63 (1H, t, J = 10.5 Hz, H-1), 6.10 (1H, d, J = 10.5 Hz, H-2), 5.15 (1H, t, J = 6.6 Hz, H-6), 2.42 (1H, t, J = 7.5 Hz, H-5), 2.27 (1H, t, J = 7.5 Hz, H-5), 2.22 (2H, m, H-4), 2.00 (6H, s, H-9), 1.68, 1.67 (3H, s, H-10), 1.63, 1.61 (3H, s, H-8), OBz [8.20 (2H, d, J = 7.7 Hz), 7.80 (1H, t, J = 7.7 Hz), 7.64 (2H, t, J = 7.7 Hz)]; ^{13}C NMR (125 MHz, acetone-d_6) δ 156.2, 156.0 (CH-1), 155.1, 155.0 (C-3), 133.2, 132.8 (C-7), 124.2, 124.1 (CH-6), 118.5, 117.7 (CH-2), 40.9 (CH_2-4), 33.4 (CH_2-5), 27.4, 26.8 (CH_3-9), 25.8, 24.7 (CH_3-10), 17.8, 17.6 (CH_3-8), OBz [164.1 (-COO-), 134.2 (CH), 130.2 (2 x CH), 129.6 (2 x CH, C)]; ESIMS *m/z* 294 $[M+Na]^+$ (100), HRESIMS *m/z* 294.1466 (calcd for $C_{17}H_{21}NO_2Na$, $[M+Na]^+$, 294.1470).

3.6.2.4. Preparation of compound ***6****.*

Following the procedure as described for oxime acylation, 4-nitrobenzoyl chloride (40 mg, 0.2 mmol), compound **2** (25.0 mg, 0.15 mmol), Et_3N (0.1 mL) and DMAP (5.2 mg) in acetone (0.5 mL), provide the desired compound **6** (1.7 mg, 3.6%). Citral-oxime 4-nitrobenzoate (**6**). ^{1}H NMR (500 MHz, acetone-d_6) δ 8.75 (1H, d, J = 10.5 Hz, H-1), 8.70 (1H, t, J = 10.5 Hz, H-1), 6.11 (1H, d, J = 10.5 Hz, H-2), 5.15 (1H, t, J = 5.2 Hz, H-6), 2.42 (1H, t, J = 7.5 Hz, H-5), 2.31-2.20 (3H, m, H-4, H-5), 1.84, 1.83 (3H, s, H-9), 1.68, 1.66 (3H, s, H-10), 1.64, 1.61 (3H, s, H-8), 4-nitrobenzoate [8.41 (2H, d, J = 8.1 Hz), 8.34 (2H, d, J = 8.1 Hz)]; ^{13}C NMR (125 MHz, acetone-d_6) δ 157.1, 156.8 (CH-1), 156.2, 156.1 (C-3), 133.3, 132.9 (C-7), 124.2, 124.1 (CH-6), 118.2, 117.3 (CH-2), 40.9 (CH_2-4), 33.4 (CH_2-5), 27.4, 26.8 (CH_3-9), 25.8, 24.7 (CH_3-10), 17.8,17.7 (CH_3-8), 4-nitrobenzoate [162.6 (-COO-), 151.8 (C), 135.6 (C), 131.6 (2 x CH), 124.7 (2 x CH)]; ESIMS *m/z* 339 $[M + Na]^+$ (100), HRESIMS *m/z* 339.1320 (calcd for $C_{17}H_{20}N_2O_4Na$, $[M+Na]^+$, 339.1321).

3.6.3. Preparation of compound ***7****.*

A suspension of citral (55.0 mg, 0.36 mmol), semicarbazide hydrochloride (334.9 mg, 3.0 mmol) and potassium carbonate (420.4 mg, 3.0 mmol) in methanol (3 mL) was stirred at 22 ºC

for 30 min. The reaction was monitored by TLC using mixture of hexane-EtOAc (8:2). Once the reaction had concluded, the methanol was evaporated, 2 mL of water was added to the residue and then was extracted by liquid-liquid partition using EtOAc (3 x 2 mL). The combined organic phases were dried over magnesium sulphate, filtering off the drying agent by gravity. The solvent was removed under reduced pressure to afford the corresponding compound **7** (71.9 mg, 95.4 %).

*3.6.4. Preparation of compound **8**.*

A solution of citral (60.7 mg, 0.4 mmol) and 4-toluenesulfonyl hydrazide (144.2 mg, 0.5 mmol) in acetonitrile (3 mL) was refluxed for 6 hours. The progress of the reaction was monitored by TLC, using a mixture of hexane-diethyl ether (5:5). Once the reaction has concluded, the solvent was removed, and the residue was purified by a silica gel CC using mixture hexanes-diethyl ether with increasing polarity (from 6:4 to 5:5) to afford the desired compound **8** (1.23 mg, 0.8 %). Citral tosylhydrazone (**8**). ^{1}H NMR (500 MHz, acetone-d_6) δ 7.92 (1H, d, *J* = 9.7 Hz, H-1), 7.91 (1H, d, *J* = 9.7 Hz, H-1), 5.82 (1H, d, *J* = 9.7 Hz, H-2), 5.07 (1H, d, *J* = 6.6 Hz, H-6), 2.21 (1H, t, *J* = 7.5 Hz, H-5), 2.12 (3H, m, H-4, H-5), 1.84 (3H, d, *J* = 1.1 Hz, H-9), 1.80 (3H, d, *J* = 1.1 Hz, H-9), 1.64, 1.58 (3H, s, H-10), 1.59, 1.55 (3H, s, H-8), tosylhydrazone [9.71 (1H, s, NH), 7.76 (2H, d, *J* = 7.9 Hz), 7.38 (2H, d, *J* = 7.9 Hz), 2.40 (3H, s)]; ^{13}C NMR (125 MHz, acetone-d_6) δ 148.9, 148.7 (C-3), 147.5, 147.3 (CH-1), 132.5 (C-7), 124.4, 124.2 (CH-6), 123.1, 122.2 (CH-2), 40.6 (CH_2-4), 33.2 (CH_2-5), 27.4, 26.8 (CH_3-9), 25.8, 24.2 (CH_3-10), 17.7, 17.1 (CH_3-8), tosylhydrazone [144.4 (C), 137.8 (C), 130.3 (2 x CH), 128.6 (2 x CH), 21.4 (CH_3)]; ESIMS *m/z* 343 $[M + Na]^+$ (100), HRESIMS *m/z* 343.1454 (calcd for $C_{17}H_{24}N_2O_2SNa$, $[M+Na]^+$, 343.1456).

*3.6.5. Preparation of compound **9**.*

A solution of citral (51.1 mg, 0.33 mmol), 2,4-dinitrophenylhydrazine (596.2 mg, 3.0 mmol) and acetic acid (20 mg, 0.3 mmol) in methanol (4 mL) was stirred at 22 ºC for 30 min. The progress of the reaction was monitored by TLC using mixture of hexane-EtOAc (8:2). The methanol was evaporated, 5 mL of water was added to the residue, and then the mixture was extracted by liquid-liquid partition using hexane (3 x 5 mL). The organic phases were concentrated under reduced pressure using a rotary evaporator, yielding the compound **9** (16.4 mg, 15.0 %). Citral 2,4-dinitrophenylhydrazone (**9**). ^{1}H NMR (500 MHz, acetone-d_6) δ 8.01 (1H, d, *J* = 10.5 Hz, H-1), 8.00 (1H, d, *J* = 10.5 Hz, H-1), 6.14 (1H, d, *J* = 9.7 Hz, H-2), 5.15 (1H, d, *J* = 5.2 Hz, H-6), 2.42 (1H, t, *J* = 7.6 Hz, H-5), 2.23 (3H, m, H-4, H-5), 1.99, 1.97 (3H, s, H-9), 1.68, 1.66 (3H, s, H-10), 1.63, 1.61 (3H, s, H-8), 2,4-dinitrophenylhydrazone [11.3 (1H, s, NH), 8.98 (1H, s), 8.64 (1H, d, *J* = 9.0 Hz), 8.36 (1H, d, *J* = 9.0 Hz)]; ^{13}C NMR (125 MHz, acetone-d_6) δ 151.9, 151.8 (C-3), 149.7, 149.4 (CH-1), 132.7 (C-7), 124.3, 123.9 (CH-6), 123.3, 122.5 (CH-2), 40.9 (CH_2-4), 33.5 (CH_2-5), 27.7, 27.0 (CH_3-9), 25.9, 25.8 (CH_3-10), 17.8, 17.5 (CH_3-8), 2,4-dinitrophenylhydrazone [145.8 (C), 138.3 (C), 130.5 (C, CH), 123.9 (CH), 117.4 (CH)]; ESIMS *m/z* 355 $[M + Na]^+$ (100), HRESIMS *m/z* 355.1380 (calcd for $C_{16}H_{20}N_4O_4Na$, $[M+Na]^+$, 355.1382).

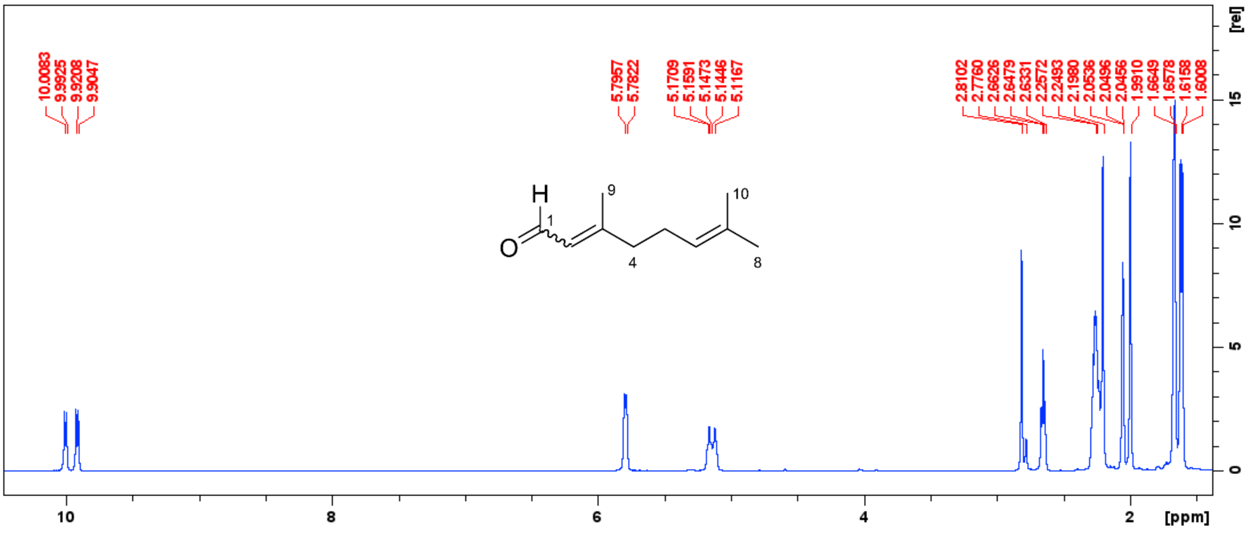

Figure S1. ^{1}H NMR spectrum [500 MHz, solvent $(CD_3)_2CO$] of compound **1**.

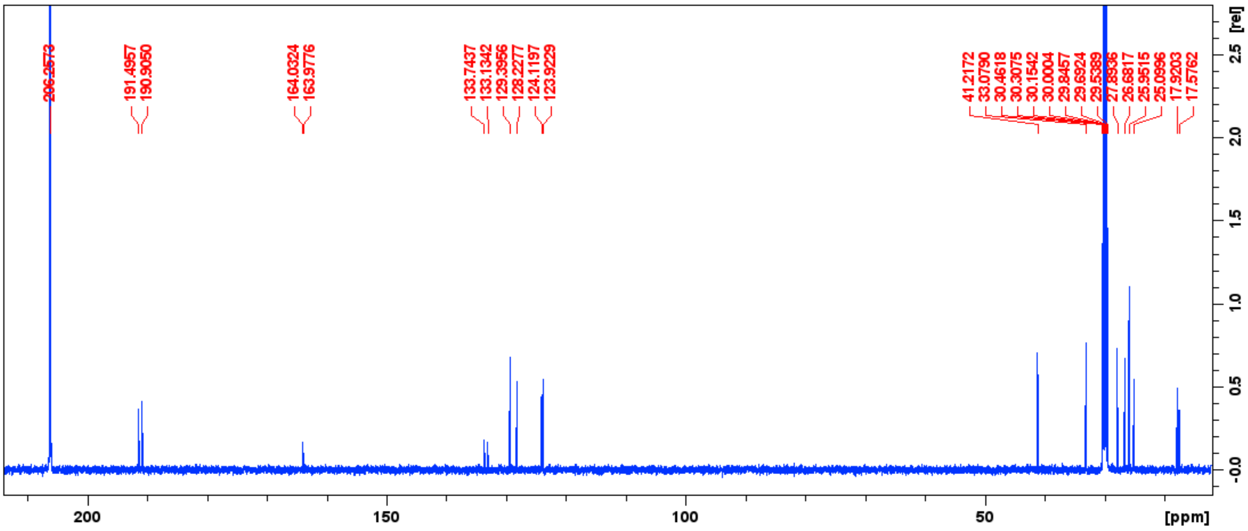

Figure S2. ^{13}C NMR spectrum [125 MHz, solvent $(CD_3)_2CO$] of compound **1**.

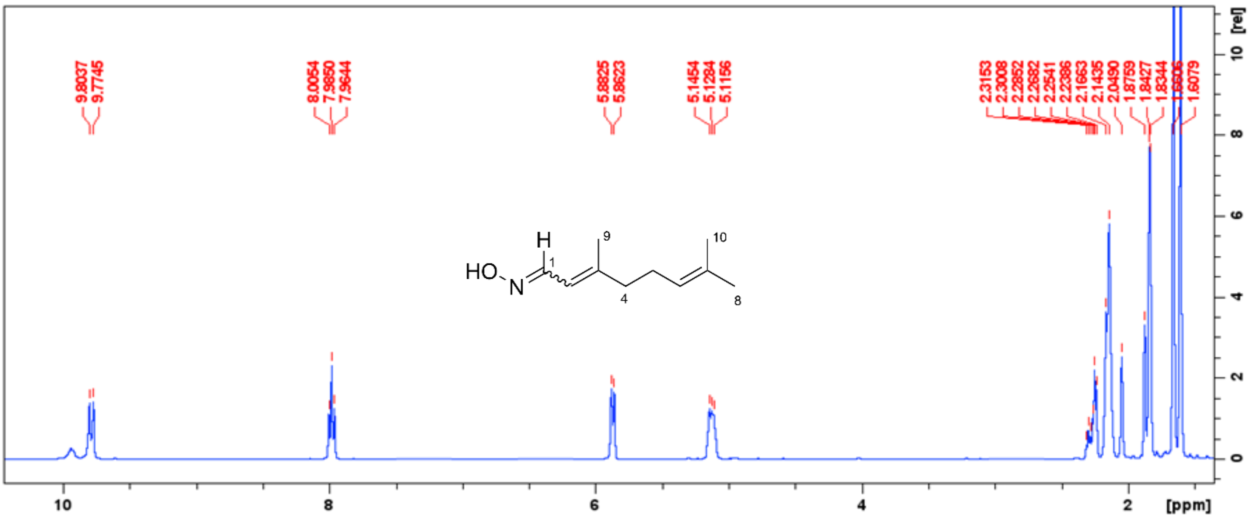

Figure S3. ^{1}H NMR spectrum [500 MHz, solvent $(CD_3)_2CO$] of compound **2**.

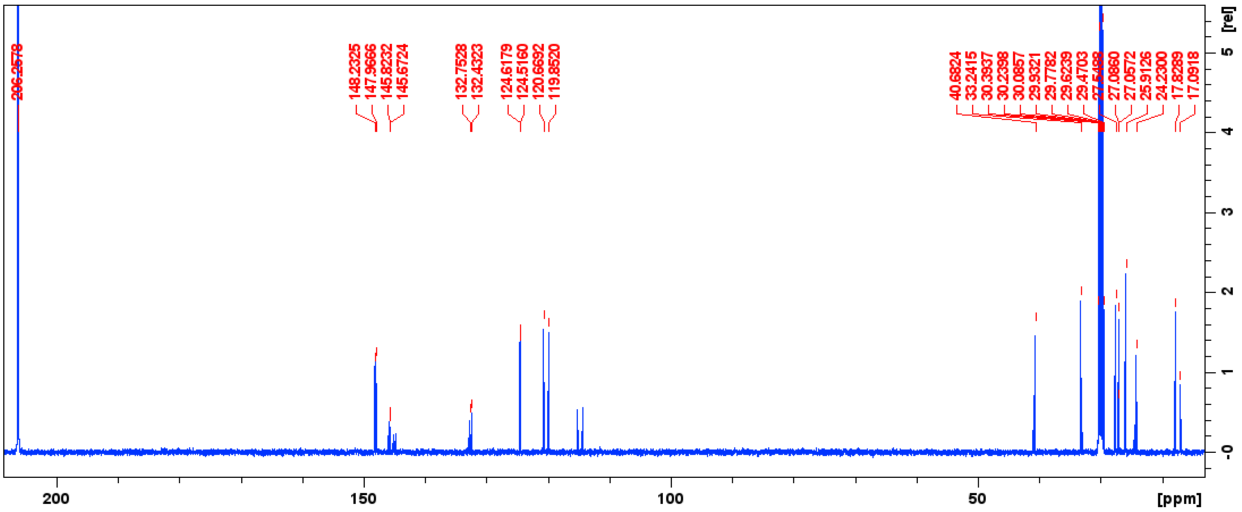

Figure S4. ^{13}C NMR spectrum [125 MHz, solvent $(CD_3)_2CO$] of compound **2**.

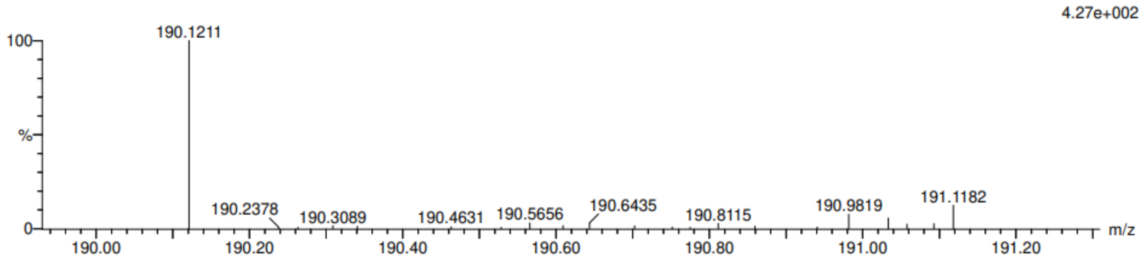

Minimum:	30.00				-1.5			
Maximum:	100.00		5.0	5.0	200.0			
Mass	RA	Calc. Mass	mDa	PPM	DBE	i-FIT	i-FIT (Norm)	Formula
190.1211	100.00	190.1208	0.3	1.6	2.5	89.5	0.0	C10 H17 N O Na

Figure S5. Mass Spectrum of compound **2**

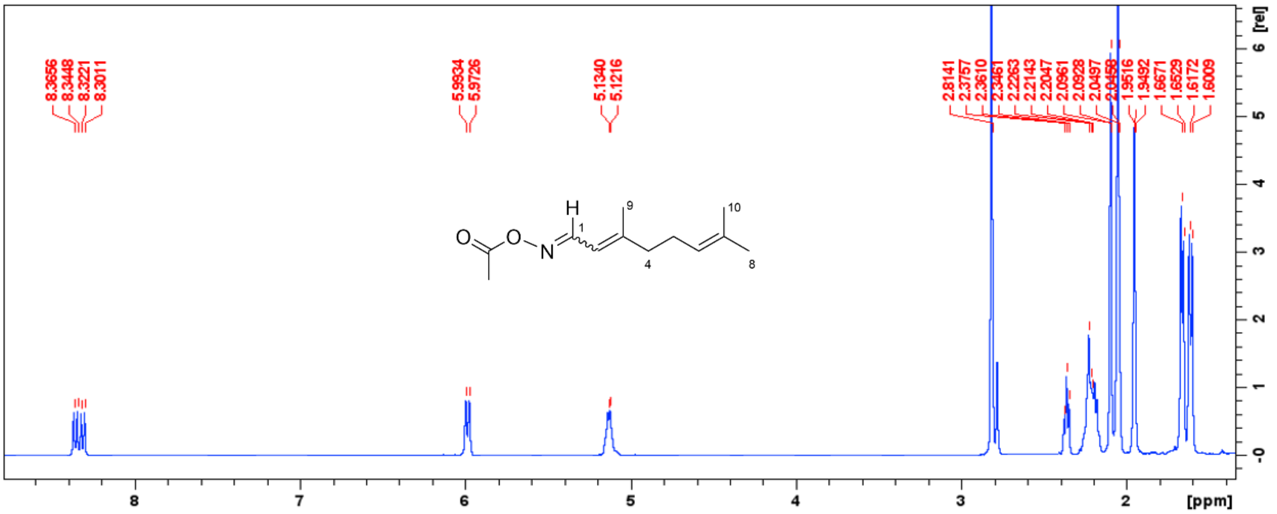

Figure S6. ^{1}H NMR spectrum [500 MHz, solvent $(CD_3)_2CO$] of compound **3**.

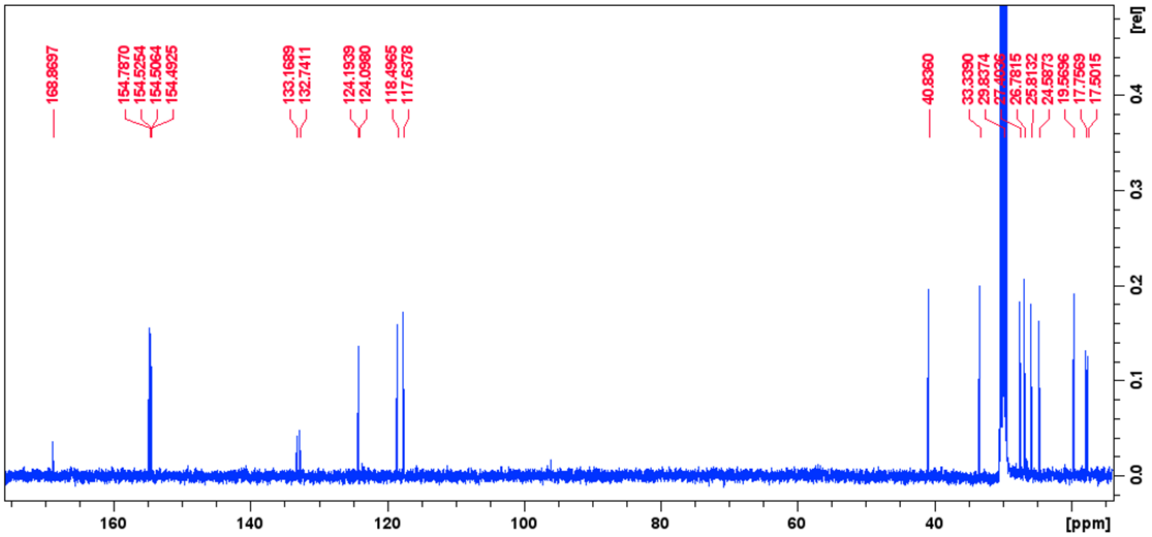

Figure S7. ^{13}C NMR spectrum [125 MHz, solvent $(CD_3)_2CO$] of compound **3**.

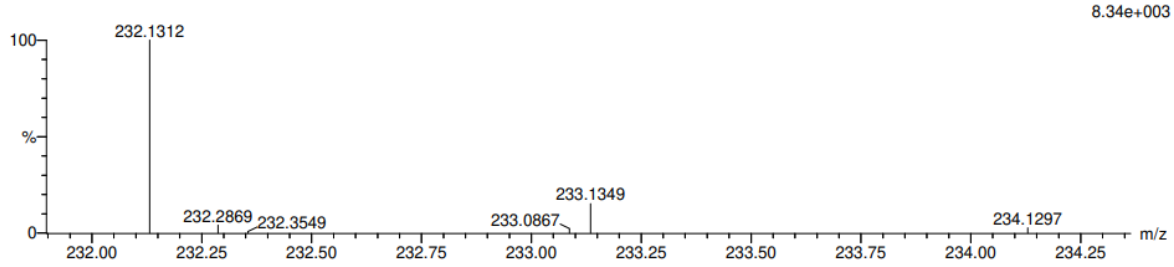

Minimum:	30.00				-1.5			
Maximum:	100.00		5.0	5.0	200.0			
Mass	RA	Calc. Mass	mDa	PPM	DBE	i-FIT	i-FIT (Norm)	Formula
232.1312	100.00	232.1313	-0.1	-0.4	3.5	46.3	0.0	C12 H19 N O2 Na

Figure S8. Mass Spectrum of compound 3

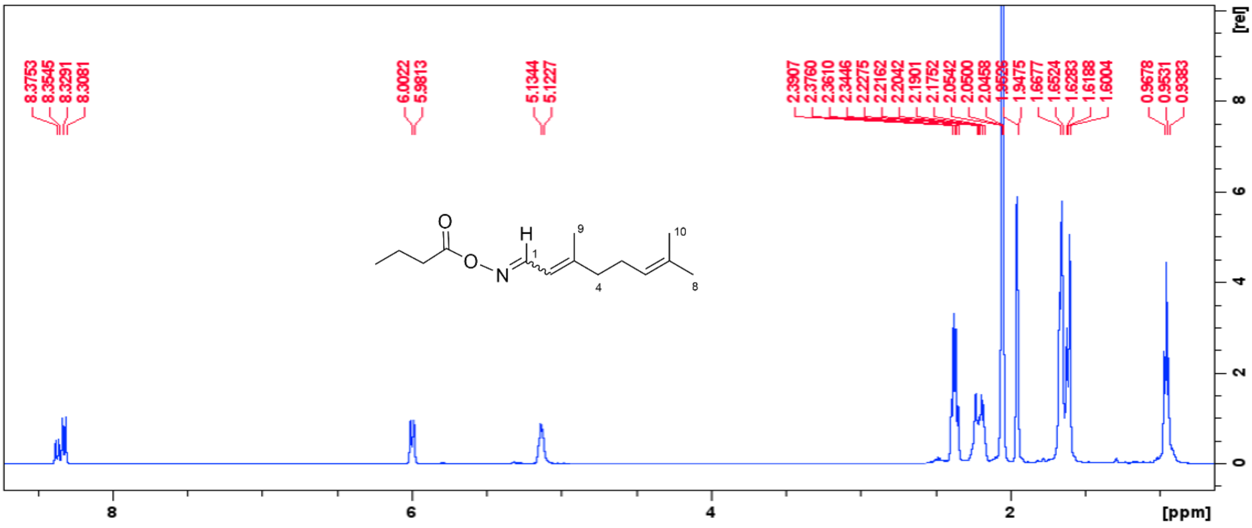

Figure S9. ^{1}H NMR spectrum [500 MHz, solvent $(CD_3)_2CO$] of compound **4**.

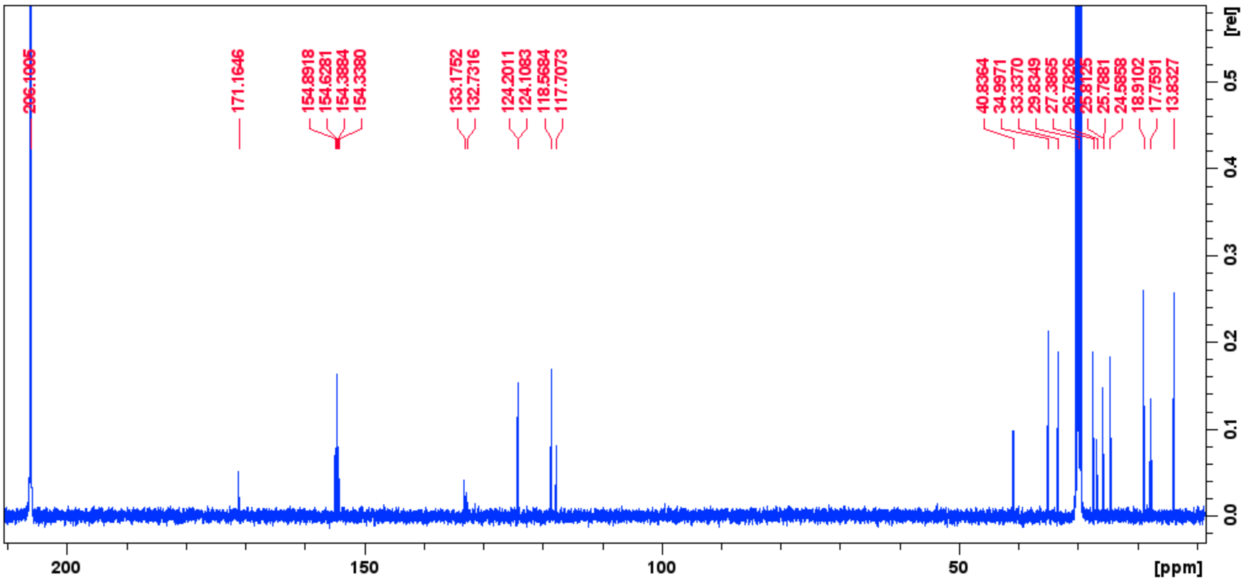

Figure S10. ^{13}C NMR spectrum [125 MHz, solvent $(CD_3)_2CO$] of compound **4**.

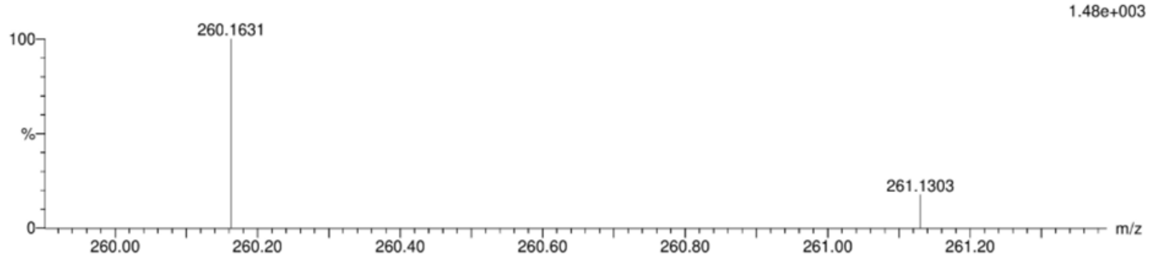

Minimum:	20.00				-1.5			
Maximum:	100.00		5.0	5.0	200.0			
Mass	RA	Calc. Mass	mDa	PPM	DBE	i-FIT	i-FIT (Norm)	Formula
260.1631	100.00	260.1626	0.5	1.9	3.5	45.9	0.0	C14 H23 N O2 Na

Figure S11. Mass Spectrum of compound **4**

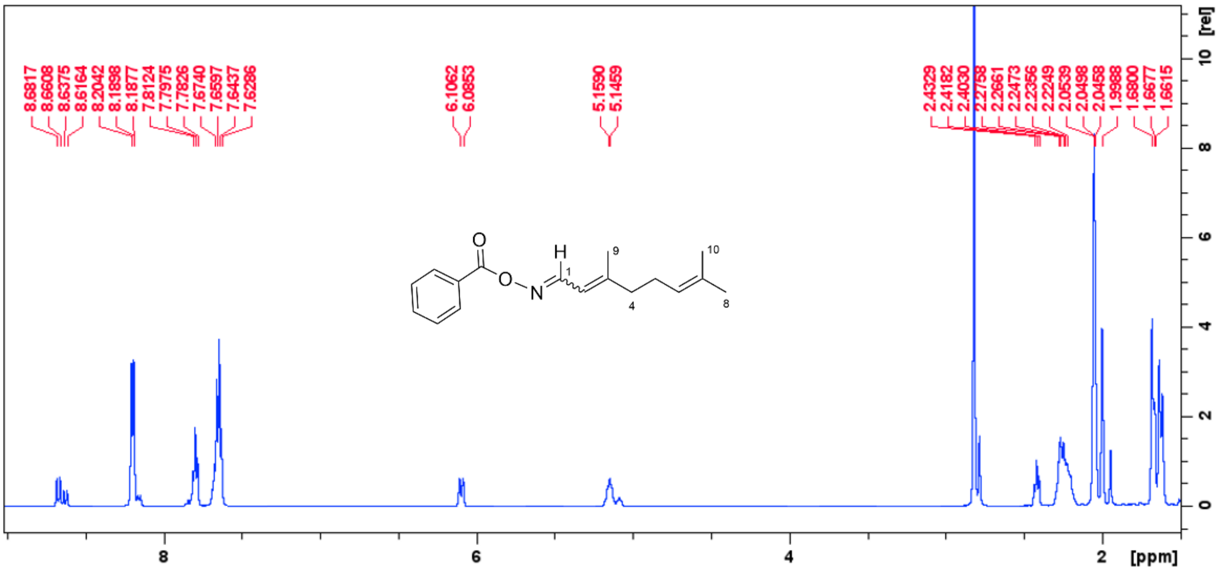

Figure S12. ^{1}H NMR spectrum [500 MHz, solvent $(CD_3)_2CO$] of compound **5**.

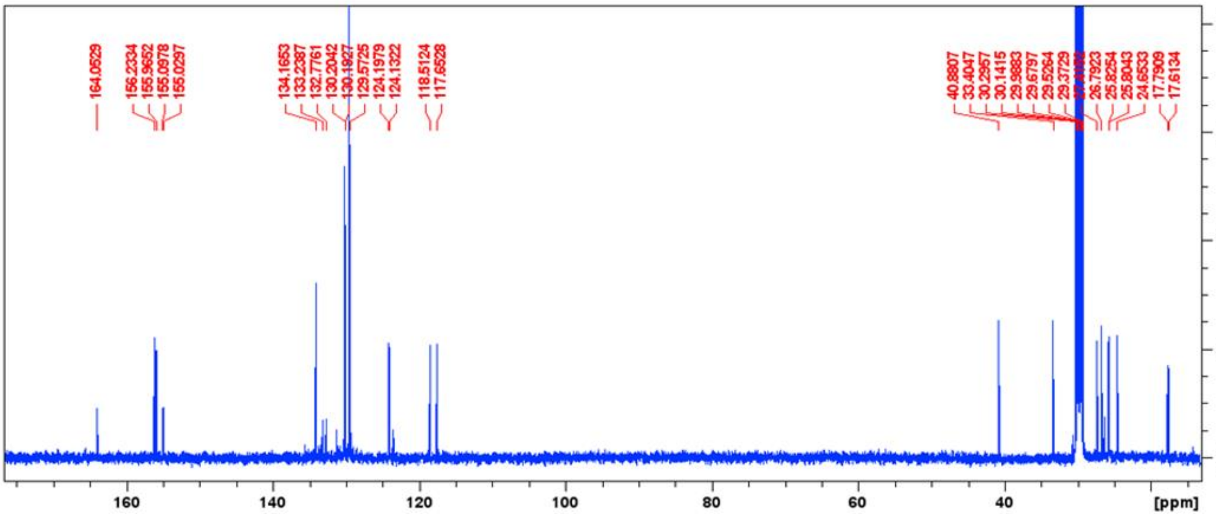

Figure S13. ^{13}C NMR spectrum [125 MHz, solvent $(CD_3)_2CO$] of compound **5**.

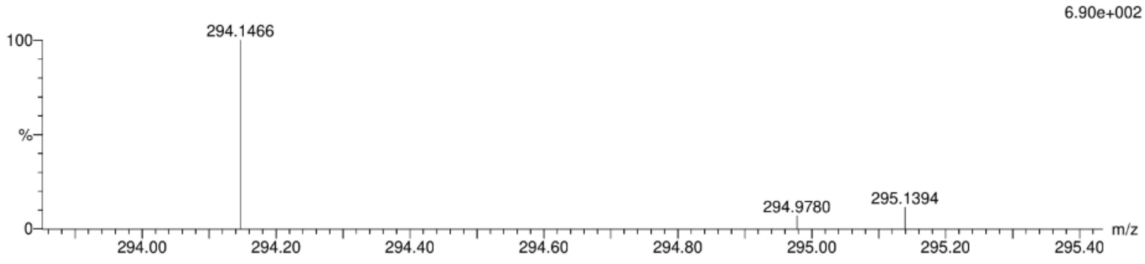

Mass	RA	Calc. Mass	mDa	PPM	DBE	i-FIT	i-FIT (Norm)	Formula
Minimum:	20.00				-1.5			
Maximum:	100.00		5.0	5.0	200.0			
294.1466	100.00	294.1470	-0.4	-1.4	7.5	43.8	1.1	C17 H21 N O2 Na
		294.1454	1.2	4.1	6.5	43.2	0.4	C14 H20 N3 O4

Figure S14. Mass Spectrum of compound 5

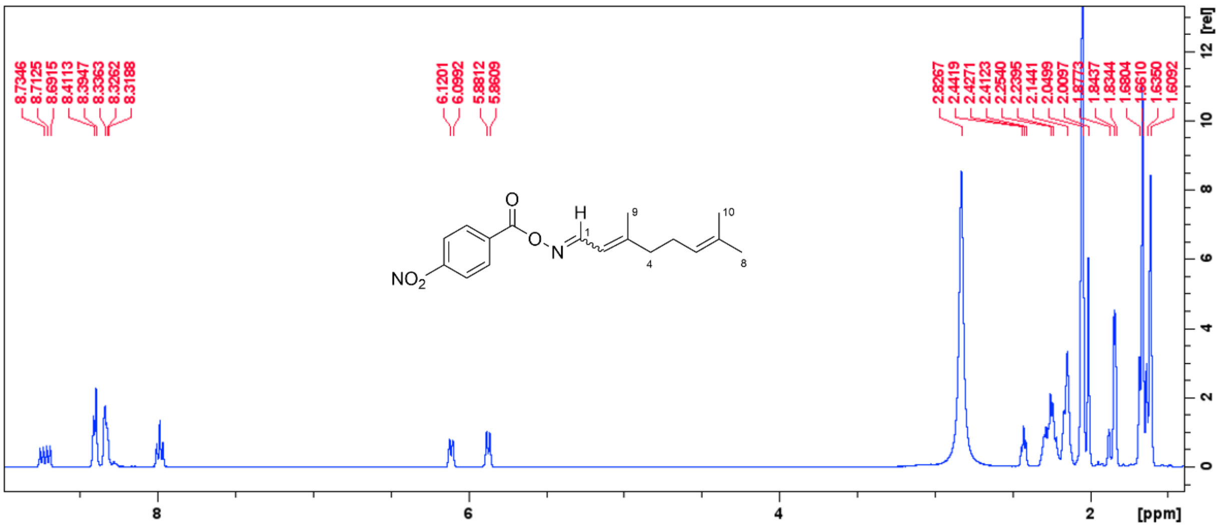

Figure S15. ^{1}H NMR spectrum [500 MHz, solvent $(CD_3)_2CO$] of compound **6**.

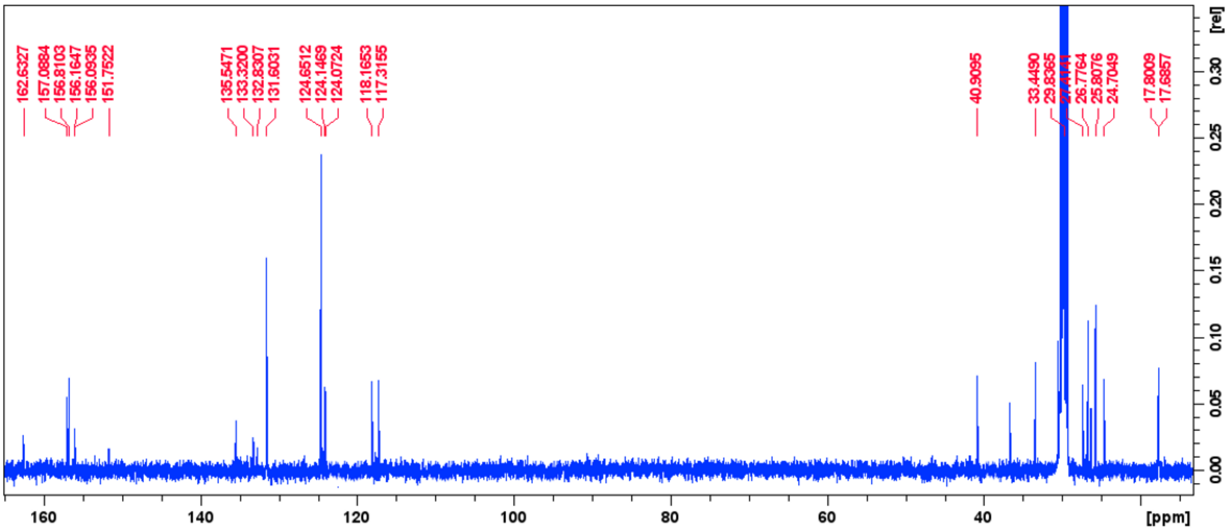

Figure S16. ^{13}C NMR spectrum [125 MHz, solvent $(CD_3)_2CO$] of compound **6**.

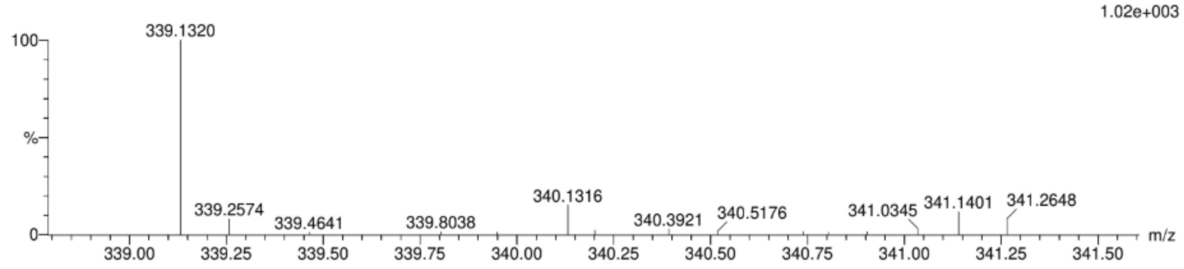

Minimum:	30.00				-1.5			
Maximum:	100.00		5.0	5.0	200.0			
Mass	RA	Calc. Mass	mDa	PPM	DBE	i-FIT	i-FIT (Norm)	Formula
339.1320	100.00	339.1321	-0.1	-0.3	8.5	89.4	0.0	C17 H20 N2 O4 Na

Figure S17. Mass Spectrum of compound **6**

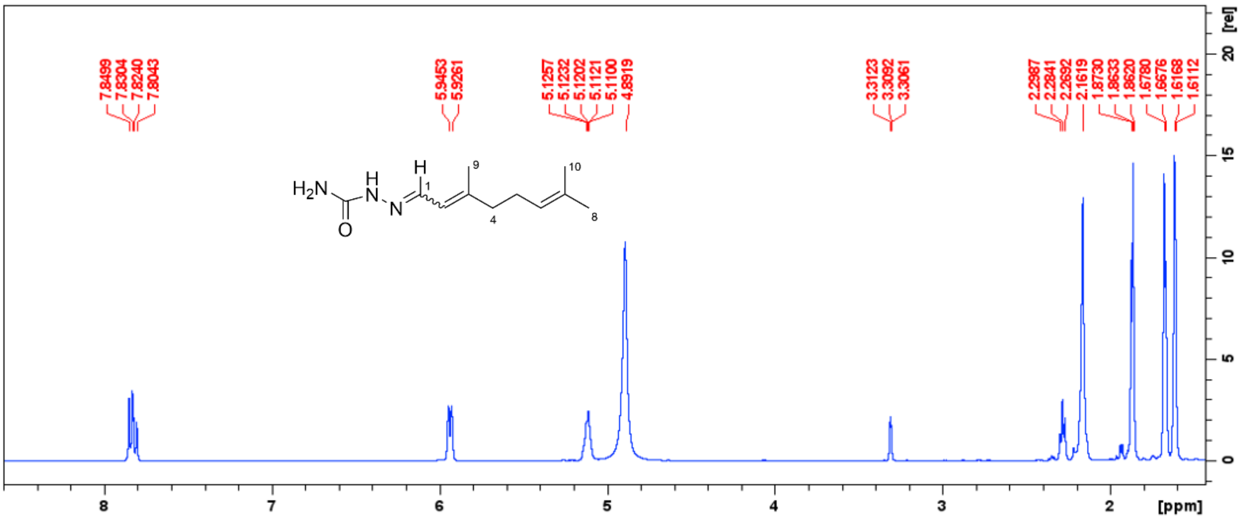

Figure S18. ^{1}H NMR spectrum [500 MHz, solvent CD_3OD] of compound **7**.

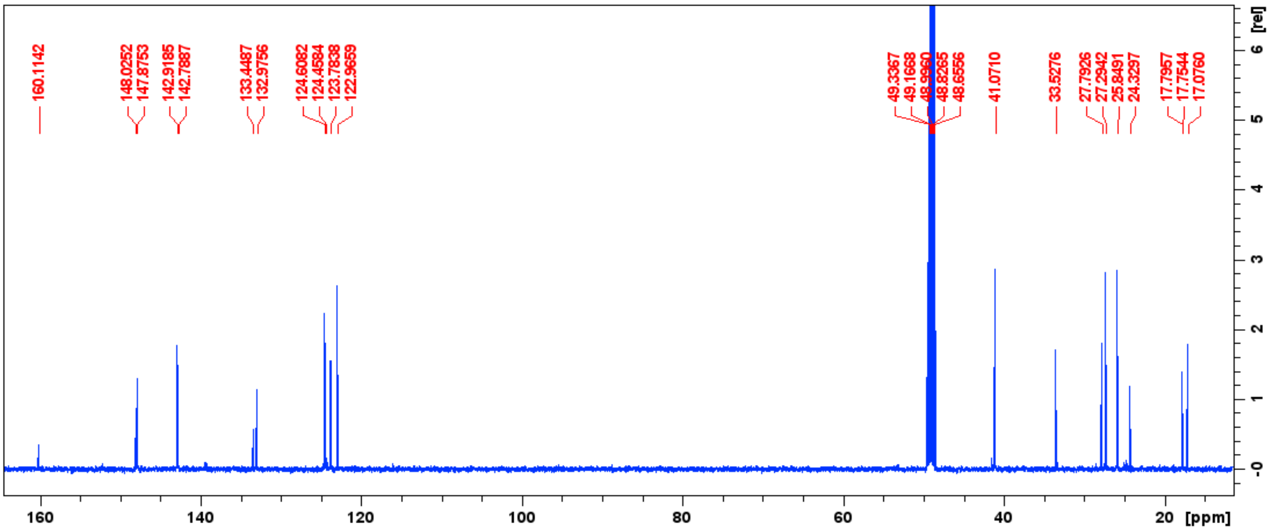

Figure S19. ^{13}C NMR spectrum [125 MHz, solvent CD_3OD] of compound **7**.

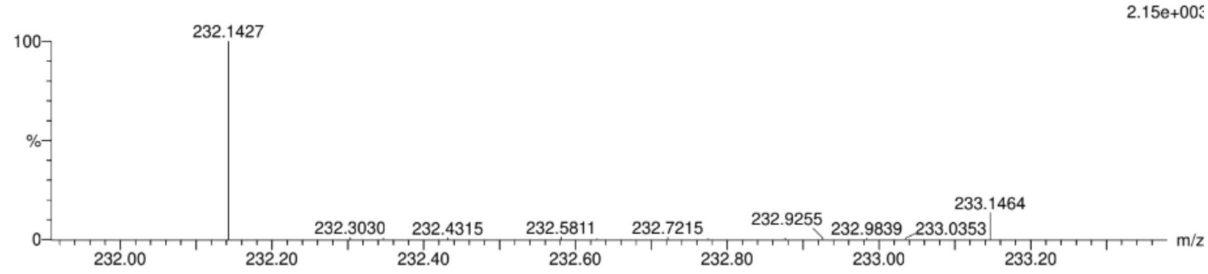

Minimum:	30.00				-1.5			
Maximum:	100.00		5.0	5.0	200.0			
Mass	RA	Calc. Mass	mDa	PPM	DBE	i-FIT	i-FIT (Norm)	Formula
232.1427	100.00	232.1426	0.1	0.4	3.5	79.0	0.0	C11 H19 N3 O Na

Figure S20. Mass Spectrum of compound **7**

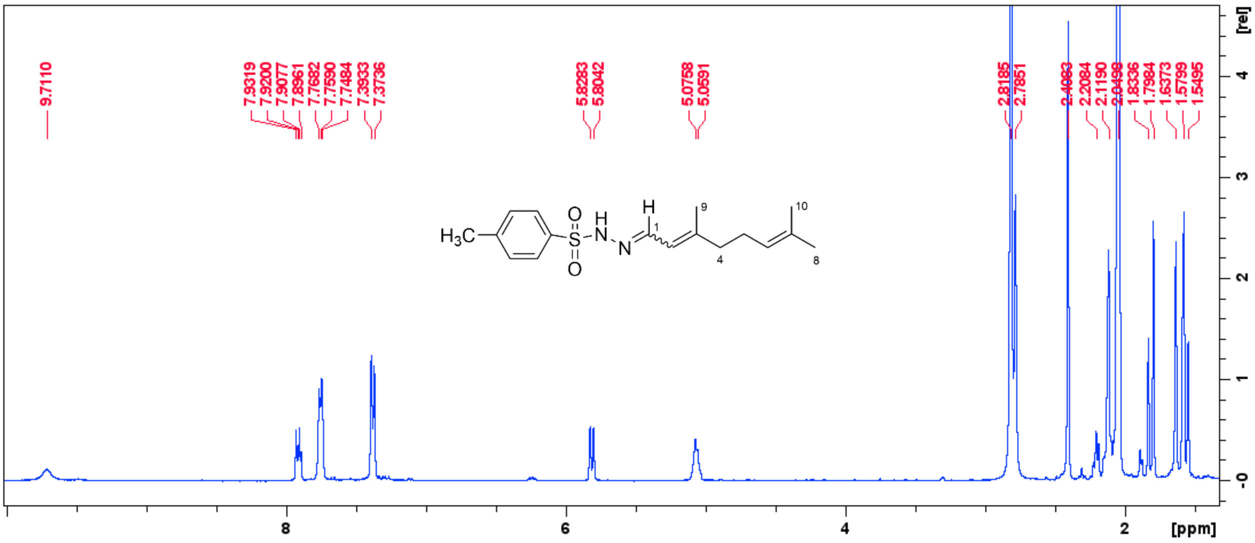

Figure S21. ^{1}H NMR spectrum [500 MHz, solvent $(CD_3)_2CO$] of compound **8**.

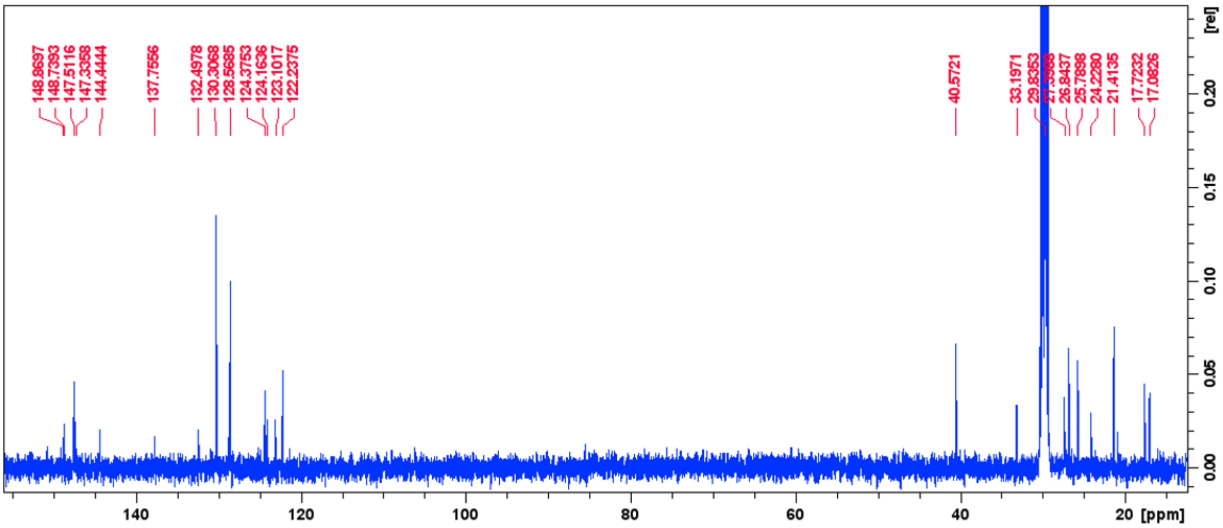

Figure S22. ^{13}C NMR spectrum [125 MHz, solvent $(CD_3)_2CO$] of compound **8**.

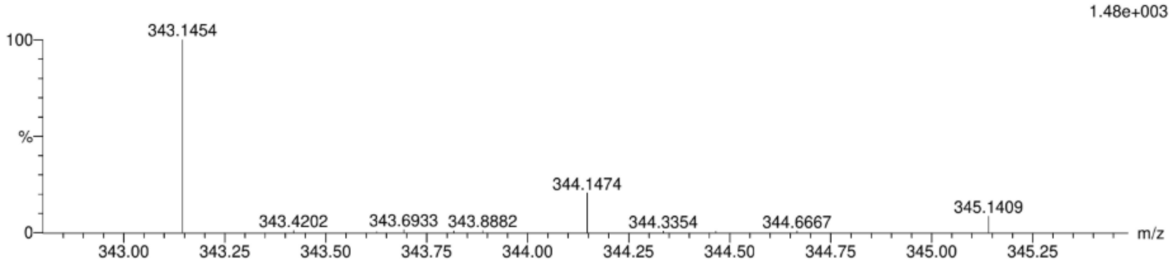

Mass	RA	Calc. Mass	mDa	PPM	DBE	i-FIT	i-FIT (Norm)	Formula
Minimum:	30.00				-1.5			
Maximum:	100.00		5.0	5.0	200.0			
343.1454	100.00	343.1456	-0.2	-0.6	6.5	49.7	0.4	C17 H24 N2 O2 Na S
		343.1440	1.4	4.1	5.5	50.3	1.1	C14 H23 N4 O4 S

Figure S23. Mass Spectrum of compound **8**

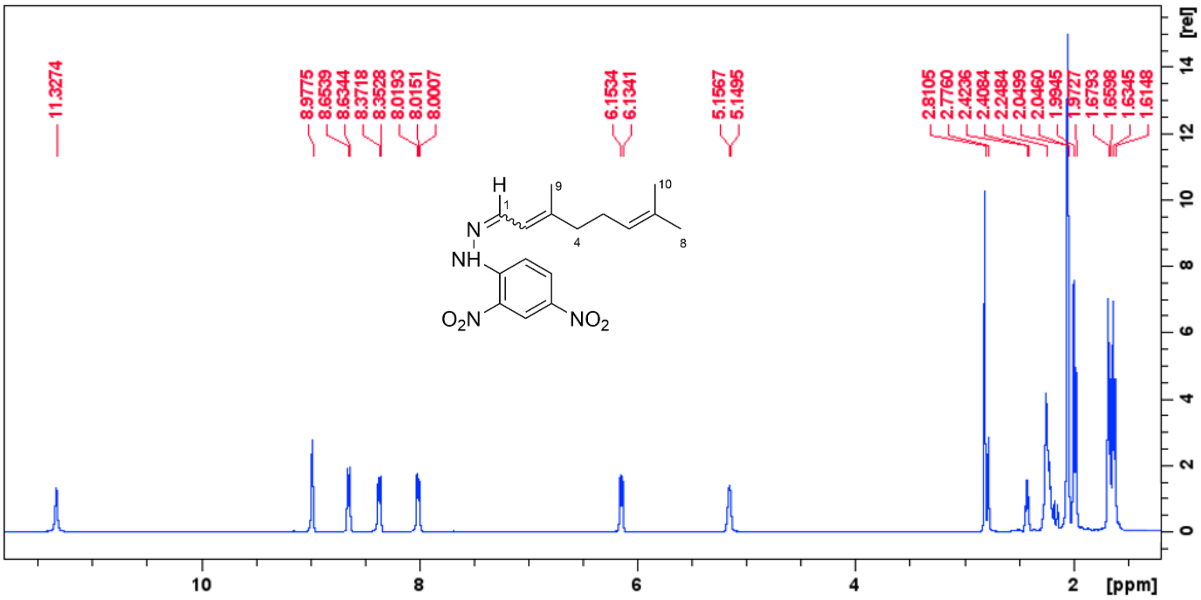

Figure S24. ^{1}H NMR spectrum [500 MHz, solvent $(CD_3)_2CO$] of compound **9**.

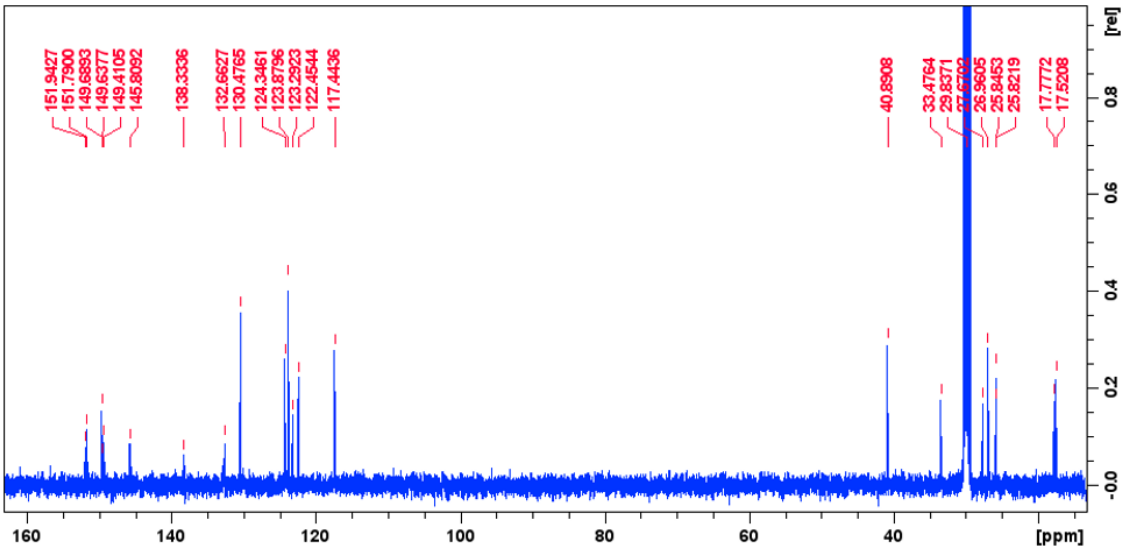

Figure S25. ^{13}C NMR spectrum [125 MHz, solvent $(CD_3)_2CO$] of compound **9**.

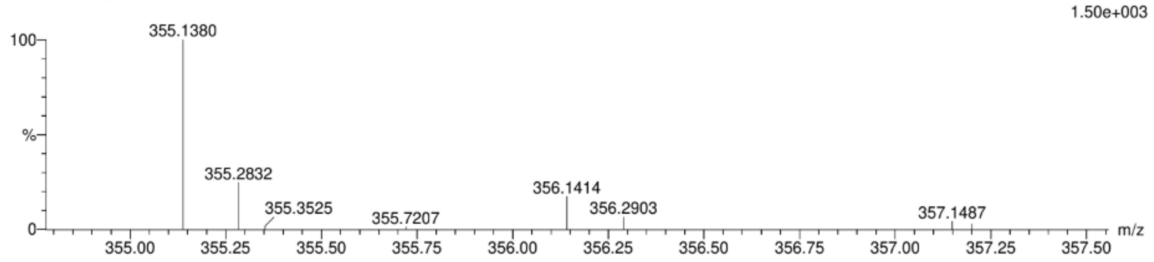

Minimum:	30.00				-1.5			
Maximum:	100.00		5.0	5.0	200.0			
Mass	RA	Calc. Mass	mDa	PPM	DBE	i-FIT	i-FIT (Norm)	Formula
355.1380	100.00	355.1382	-0.2	-0.6	8.5	50.2	0.0	C16 H20 N4 O4 Na

Figure S26. Mass Spectrum of compound **9**
